# Amorphization activated ruthenium-tellurium nanorods for efficient water splitting

**DOI:** 10.1038/s41467-019-13519-1

**Published:** 2019-12-12

**Authors:** Juan Wang, Lili Han, Bolong Huang, Qi Shao, Huolin L. Xin, Xiaoqing Huang

**Affiliations:** 1 College of Chemistry, Chemical Engineering and Materials Science, Soochow University, 215123 Jiangsu, China; 2 Department of Physics and Astronomy, University of California, Irvine, CA 92697 USA; 3 Department of Applied Biology and Chemical Technology, The Hong Kong Polytechnic University, Hung Hom, Kowloon, Hong Kong SAR

**Keywords:** Electrocatalysis, Solid-state chemistry, Computational chemistry

## Abstract

Pursuing active and durable water splitting electrocatalysts is of vital significance for solving the sluggish kinetics of the oxygen evolution reaction (OER) process in energy supply. Herein, theoretical calculations identify that the local distortion-strain effect in amorphous RuTe_2_ system abnormally sensitizes the Te-pπ coupling capability and enhances the electron-transfer of Ru-sites, in which the excellent inter-orbital p-d transfers determine strong electronic activities for boosting OER performance. Thus, a robust electrocatalyst based on amorphous RuTe_2_ porous nanorods (PNRs) is successfully fabricated. In the acidic water splitting, a-RuTe_2_ PNRs exhibit a superior performance, which only require a cell voltage of 1.52 V to reach a current density of 10 mA cm^−2^. Detailed investigations show that the high density of defects combine with oxygen atoms to form RuO_x_H_y_ species, which are conducive to the OER. This work offers valuable insights for constructing robust electrocatalysts based on theoretical calculations guided by rational design and amorphous materials.

## Abstract (web summary)

Elctrochemical water splitting is of vital significance for energy conversion and storage. Here the authors show an electrocatalyst based on amorphous ruthenium-tellurium porous nanorods which exhibit significantly improved OER performance than its crystalline counterparts

## Introduction

Electrochemical water splitting, emerging as an attractive technology for generating hydrogen (H_2_) and oxygen (O_2_), provides a potential to address environmental degradation and energy crises due to its pure and clean products^1–4^. Noble-metal Pt and Ir/Ru are considered to be the benchmark catalysts for the hydrogen evolution reaction (HER) and the oxygen evolution reaction (OER), respectively^5–7^. However, a high voltage is still needed to drive the reaction process because of the sluggish kinetics for OER, especially in acidic conditions^8–11^. Significant efforts have been undertaken to design novel catalysts to overcome this obstacle, including heteroatoms doping, functionalization, and so on^12–16^. Although much progress in developing bifunctional electrocatalysts for alkaline water splitting has been realized, the overpotential and corrosion resistance are still far from satisfactory under harsh acidic conditions, which hamper the development of proton exchange membrane water electrolyzers^17–19^.

Recent studies have been primarily focused on enhancing the intrinsic activity to improve catalytic efficiency, such as phase and interface engineering, creating grain boundaries and regulating electronic structures^20–24^. It should be noteworthy that those resulting catalysts are overwhelmingly based on crystalline materials and tend to ignore their amorphous counterparts. Amorphous materials are generally entities, in which the arrangement of their internal atoms is not periodic but only bear the local short-range order. The inherent disorderliness of amorphous materials can produce abundant “dangling bonds” and defects in the loosely bonded atomistic free-volume zones, which can provide more active sites and thus improve catalytic activity^25–27^. In addition, the unique structure and isotropic properties endow amorphous materials strongly corrosion resistance in both acidic and alkaline conditions, providing fresh insights into the search for highly stable catalysts^28,29^. For example, Zhang et al. prepared amorphous lithium-incorporated palladium phosphosulfide nanodots (Li-PPS NDs) by electrochemically lithiated layered Pd_3_P_2_S_8_ crystal. Interestingly, this amorphization process can activate the electrochemically inert Pd_3_P_2_S_8_, thereby significantly enhances its HER activity^30^. The obtained amorphous Li-PPS NDs also possess excellent stability under the acidic condition that the decay of current density is negligible after 10000 potential cycles. Inspired by the above, catalyst designs based on amorphous materials are therefore an attractive strategy for developing highly active and stable electrocatalysts for water splitting under harsh environments.

Herein, guided by the theoretical mechanism study of the intrinsic high electroactivity revealed in the amorphous structure, the a-RuTe_2_ porous nanorods (a-RuTe_2_ PNRs) with bullet-like outline have been designed and synthesized as robust water splitting electrocatalysts. Density functional theory (DFT) calculations reveal that high amorphization degree renders the local short-range disorder to be distinguished, which inevitably induces distortion-strain effect (DS) and thus leads the system to be the meta-stable state. Such energetic trend not only facilitates the variation of local Te-coordination for flexible bonding but also induces a medium-to-long range pπ coupling to efficiently annihilate the notorious crystal-field-splitting effect of Ru for highly active intra- and inter-orbital electron-transfer. From the view of strong electron-lattice coupling effect, the short-range disorder contributes to the intrinsic guarantee for high OER activities within pH-universal conditions. As an electrocatalytic result, the a-RuTe_2_ PNRs present a significantly improved OER performance than its crystalline counterparts in harsh environments, especially in 0.5 M H_2_SO_4_. In detail, it is capable of delivering an overpotential of as low as 245 mV for OER, far better than those of crystalline RuTe_2_ PNRs (c-RuTe_2_ PNRs) and the benchmark electrocatalyst Ir/C. By constructing a-RuTe_2_ PNRs as a two-electrode system in the acidic electrolyte, only a cell potential of 1.52 V is needed to generate 10 mA cm^−2^. Experimental results demonstrate that the distorted Ru-Te bond can be derived from the high density of defects. Additionally, the RuO_x_H_y_ species form by combining the defect with oxygen atoms also contribute to the reaction process.

## Results

### DFT theoretical simulations

**Fig. 1:**
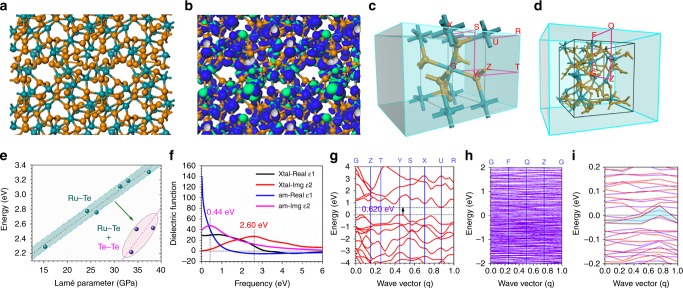
Electronic structures comparison between the crystalline and amorphous RuTe_2_. **a** Local atomic configurations for the amorphous RuTe_2_. **b** The real spatial contour plots for bonding and anti-bonding orbitals near E_F_. **c** The Brillouin zone of the crystalline RuTe_2_, in which the G, Z, T, Y, S, X, U, and R denote high-symmetry points within the reciprocal space (red). **d** The Brillouin zone of the amorphous RuTe_2_, in which the G, F, Q, and Z denote high-symmetry points within the reciprocal space (red). **e** Structural formation energy variation trend with related to the Lamé parameters for describing the bulk lattice distortion induced strain effect, as well as the Te-Te bond formation. **f** The dielectric function comparison. **g** The band structure of the crystalline RuTe_2_ and **h** the amorphous RuTe_2_. **i** Enlarged display of the amorphous RuTe_2_ band structure.

Although strain effect has been utilized to enhance the electron transfer activity to facilitate the electrocatalysis, the corresponding characterization still remains a huge challenge in the amorphous materials. In order to investigate the intrinsic activity of amorphous samples, we have carried out DFT calculations to evaluate the OER process of amorphous RuTe_2_ system before experiments. From the model, the weighted average coordination (CN) of Ru is lowered (CN = 6 for crystalline RuTe_2_) staying between 4 (tetrahedral) and 6 (octahedral), while Te sites are more flexible ranged from CN = 2 to CN = 6 with Te-Te bonds (Fig. 1a). The bonding and anti-bonding orbitals near the Fermi level (E_F_) demonstrate a p-π electron-rich character given by Te-sites, indicating the high electronic sensitivities of Te to couple O-2p orbital for H_2_O activation (Fig. 1b). The line-up of reciprocal, highly symmetrical points in both crystalline and amorphous structures indicates the different electron transfer paths within the Brillouin zone, supporting the distinct electron transfer ability (Fig. 1c, d). As short-range Ru-Te disordered, the intrinsic DS strengthens the driving force on the electronic activities. The Lamé parameters reflect the correlation within homogenous, isotropic, and continuum medium. With increasing DS-effect, the instabilities of Ru-Te bonding environment are enlarged. However, as the energetic trend reflects, Te-Te homopolar bond formation plays a key role in facilitating wider-range relaxations towards higher stabilities, supporting the dominant role of the highly sensitive p-π coupling in both energetic performance and electronic activities (Fig. 1e). Moreover, the dielectric function of the amorphous structure is obviously larger than the crystalline structure, especially the static dielectric function ε_1_. The imaginary part ε_2_ of the dielectric function of the amorphous reaches the first peak value at 0.440 eV, which is much smaller than 2.60 eV of the crystalline, confirming the much smaller electron transition from the top of valence band to the bottom of conduction band near the reciprocal, highly symmetrical points (Fig. 1f).

**Fig. 2:**
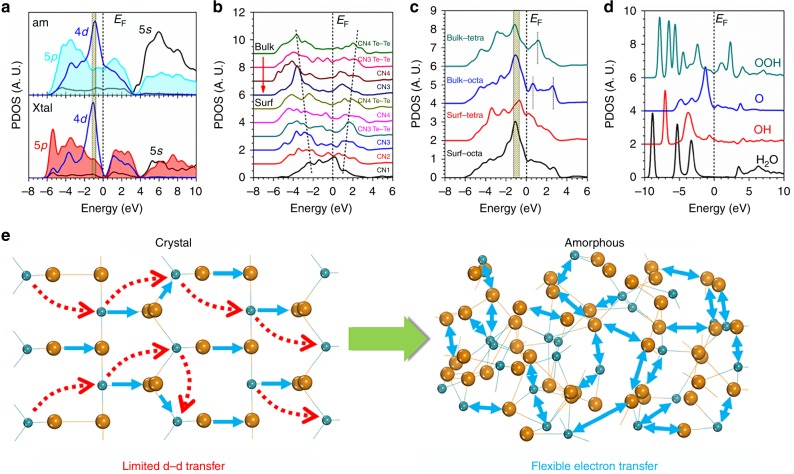
Electronic activities of the amorphous RuTe_2_ for OER. **a** PDOSs comparison between amorphous and crystalline RuTe_2_ systems. **b** Site-dependent PDOSs variation trend of Te-5p band. **c** Site-dependent PDOSs variation of Ru-4d band. **d** Individual PDOSs contributions of O-species from OER process. **e** Schematic diagram of the electronic activity enhancement in amorphous structure.

To further distinguish the intrinsic difference in electronic contribution of the crystalline and amorphous structures in boosting the catalysis, the band structures are plotted. The detailed theoretical derivation and discussion of the intrinsic electronic contribution enhancement of the amorphous structure in catalysis is provided in the Supplementary Note 1. Notably, the crystalline structure shows an evident indirect band gap of 0.620 eV, which demonstrates the energy barriers for *d-d* and *d-p* transitions (Fig. 1g). In comparison, the amorphous RuTe_2_ exhibits electron-rich feature crossing the Fermi level without any energy gap, supporting the facile *d-d* electron transition for achieving superior OER performance. Moreover, the forbidden *p-d* electron transition in the crystalline structure has been loosen by the amorphous structure due to the induced intrinsic DS effect (Fig. 1h, i). Therefore, these results indicate that such different electron transfer leads to the contrast selectivity of OER process. Meanwhile, the projected partial density of states (PDOSs) reveal that Ru-4d-t_2g_ activity (valence-peak) and valence-band-centers (*E*_V_ − 1.2 eV and *E*_V_ − 1.0 eV for amorphous and crystal system respectively, *E*_V_ = 0 for E_F_) remain while the DS-effect relaxes the forbidden rule of intra-orbital e_g_-t_2g_ electron-transfer, which annihilates the gap between the e_g_-t_2g_ splitting of the Ru-4d band in the amorphous structure. The distorted Ru-Te lattice increases the homogeneity for efficient inter-d-orbital electron-transfer ability among Ru sites (Fig. 2a). The surface Te-5p dominantly occupies from *E*_V_ − 4.5 eV to *E*_F_ with the most overlapping with Ru-4d, enhancing the activities of p-d coupled electron-exchange. The stronger electron transfer between p-d than the p-p also supports the anti-oxidation of the RuTe_2_. The enhanced bonding and anti-bonding splitting effect of Te-5p band in the bulk region with higher CN results in gradually inert and less overlapped with Ru-4d (Fig. 2b). The surface Ru-4d band obviously merges the electronic occupations, resulting in the annihilation of the e_g_-t_2g_ splitting character on both surfaces octahedral and tetrahedral bonding sites. Therefore, the surface distorted Ru-Te bonding minimizes the crystal-field splitting effect for the surface Ru-sites (Fig. 2c). The p-π orbital of H_2_O exhibits a substantial overlapping with Te-5p bands showing a strong p-π coupling to activate the H_2_O molecule for efficient splitting. From OH to O, the p-π coupling becomes stronger with the increase of p-orbital electronic activities. Further on the OOH, a slightly weaker electronic activity is demonstrated due to the hydrogenation effect for O-pπ passivation (Fig. 2d). The schematic diagram of the electron transfer pathways has been presented to show the enhanced electronic activity of the amorphous structure of RuTe_2_ in promoting the catalysis (Fig. 2e).

**Fig. 3:**
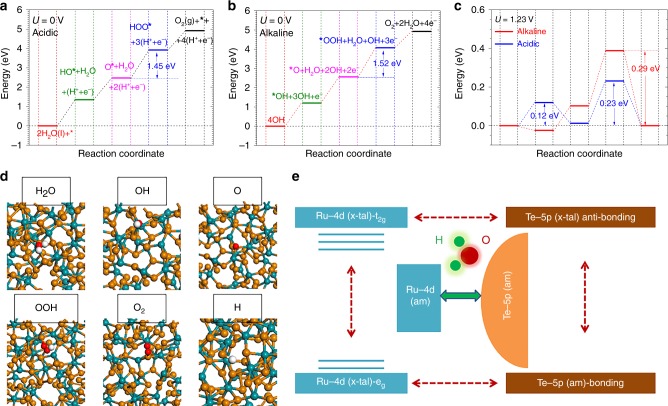
OER energy profile and activity plot. **a** The free energetic pathways for acidic OER at *U* = 0 V. The corresponding reaction mechanism in the acidic is shown in the plot and calculation setup. **b** The alkaline OER pathway at *U* = 0 V. The different reaction mechanisms with the acidic OER based on key adsorbates and co-reactants is supplied in the plot and calculation setup. **c** The OER pathways of acidic and alkaline conditions at *U* = 1.23 V. **d** Local structural configurations of initial reactant, intermediates or final product on the amorphous RuTe_2_ surface. **e** Schematic diagram for illustrating the amorphous RuTe_2_ boosting the OER performance via highly efficient electron-transfer site-independently.

We move onto the OER pathways (Fig. 3a–c). In acidic condition, the Ru-Te surface performs an excellent H_2_O splitting with the substantially low activation energy for [*OH+H^+^+e^−^]. The secondary de-hydrogenation performs at even lower energetic level. This trend guarantees the potential determining step to form OOH^-^ favoring at a lower energy of 3.92 eV relative to initial H_2_O level (Fig. 3a). In alkaline condition, the favorable bonding of both OH and O facilitates the OOH formation with a lower barrier of 1.52 eV (Fig. 3b). Under the *U* = 1.23 V potential, the overpotentials (i.e. max{[barrier-1.23 eV]/e}) are 0.23 V (acidic) and 0.29 V (alkaline), respectively (Fig. 3c). From local structure perspectives, the H_2_O stabilizes between the Ru and Te sites, while OH, O, and OOH uniquely adsorbs on distorted Te sites, where the local Te-CN flexibly varied (Fig. 3d). We confirm that OER performance originates from surface DS-effect, which sensitizes Te-pπ coupling and annihilates the crystal-field splitting effect of Ru for highly active intra- and inter-orbital electron-transfer (Fig. 3e).

### Catalyst synthesis and characterization

**Fig. 4:**
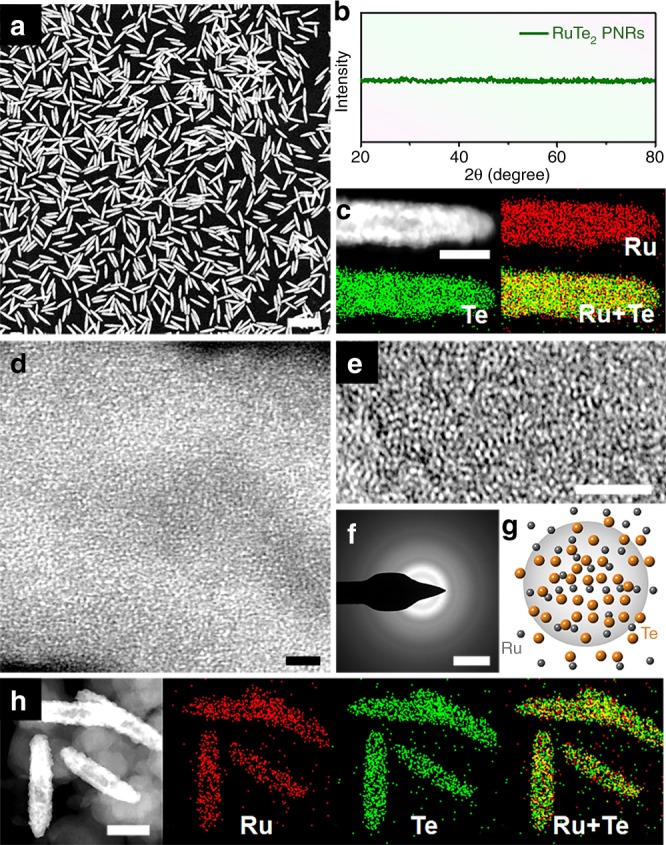
Morphology and composition profile analysis. **a** HAADF-STEM image, **b** XRD pattern, **c** STEM-ADF image and EDS elemental mappings of RuTe_2_ PNRs. **d** Atomic-resolution HAADF-STEM image, **e** HRTEM image, **f** SAED pattern, **g** structural model, and **h** STEM-ADF image and EDS elemental mappings of carbon supported a-RuTe_2_ PNRs. Scale bar, **a** 200 nm; **c** 10 nm; **d**, **e** 2 nm; **f** 5 1/nm, and **h** 40 nm.

To realize the highly electroactive amorphous surface for OER as the theoretical predictions, we present the fabrication strategy with the rational design by following the guidance of the DFT calculations. We have successfully designed, prepared and compared RuTe_2_ PNRs in both amorphous and crystalline conditions. The pristine amorphous RuTe_2_ PNRs were prepared by a simple hydrothermal process, in which potassium tellurite (K_2_TeO_3_) and hexaammineruthenium (III) chloride (Cl_3_H_18_N_6_Ru) were used as metal precursors^31^. High-angle annular dark-field scanning transmission electron microscopy (HAADF-STEM) image shows that the products exhibit one-dimensional (1D) nanorods structure with high dispersion (Fig. 4a). TEM images show that the nanorods have a porous structure and bullet-like profile with the average diameter and length of 17.0 ± 1.8 nm and 99.7 ± 5.8 nm, respectively (Supplementary Fig. 1). The Ru/Te molar ratio was determined to be around 1:2 by inductively coupled plasma atomic emission spectrometry (ICP-AES) and scanning electron microscopy energy-dispersive X-ray spectroscopy (SEM-EDS). No significant diffraction peaks were observed by powder X-ray diffraction (PXRD) (Fig. 4b), demonstrating that the RuTe_2_ PNRs are amorphous, corresponding to the high-resolution TEM (HRTEM) and selected area electron diffraction (SAED) (Supplementary Fig. 2). STEM-EDS element mappings reveal that the Ru and Te are evenly distributed along the RuTe_2_ PNRs (Fig. 4c). Subsequently, the RuTe_2_ PNRs were loaded on VC-X72 carbon (Supplementary Fig. 3) and then thermally treated under air atmosphere^32,33^, where only a broad carbon peak can be found in their XRD pattern, demonstrating that the RuTe_2_ PNRs still maintain an amorphous structure (denoted as a-RuTe_2_ PNRs) (Supplementary Fig. 4). HAADF-STEM and TEM images show that the PNRs keep 1D porous nanorod profile with a slight increase in diameter and length. As revealed by the atomic-resolution HAADF-STEM image, the atoms exhibit a random distribution without significant periodicity, proving the structural disorder of a-RuTe_2_ PNRs (Fig. 4d). Moreover, only blurred areas with no clear long-range ordered atomic arrangements can be observed from HRTEM, further demonstrating the amorphous feature of a-RuTe_2_ PNRs (Fig. 4e). SAED pattern also reveals a set of distinct rings composed of diffraction spots (Fig. 4f), being in agreement with the XRD results. The structural model of RuTe_2_ PNRs with the amorphous feature was successfully described (Fig. 4g), where the Ru and Te atoms exhibit a random arrangement with isotropic, short-range order in atomistic free-volume zones. STEM-EDS element mappings reveal that the Ru and Te are still distributed uniformly throughout the whole PNRs (Fig. 4h).

**Fig. 5:**
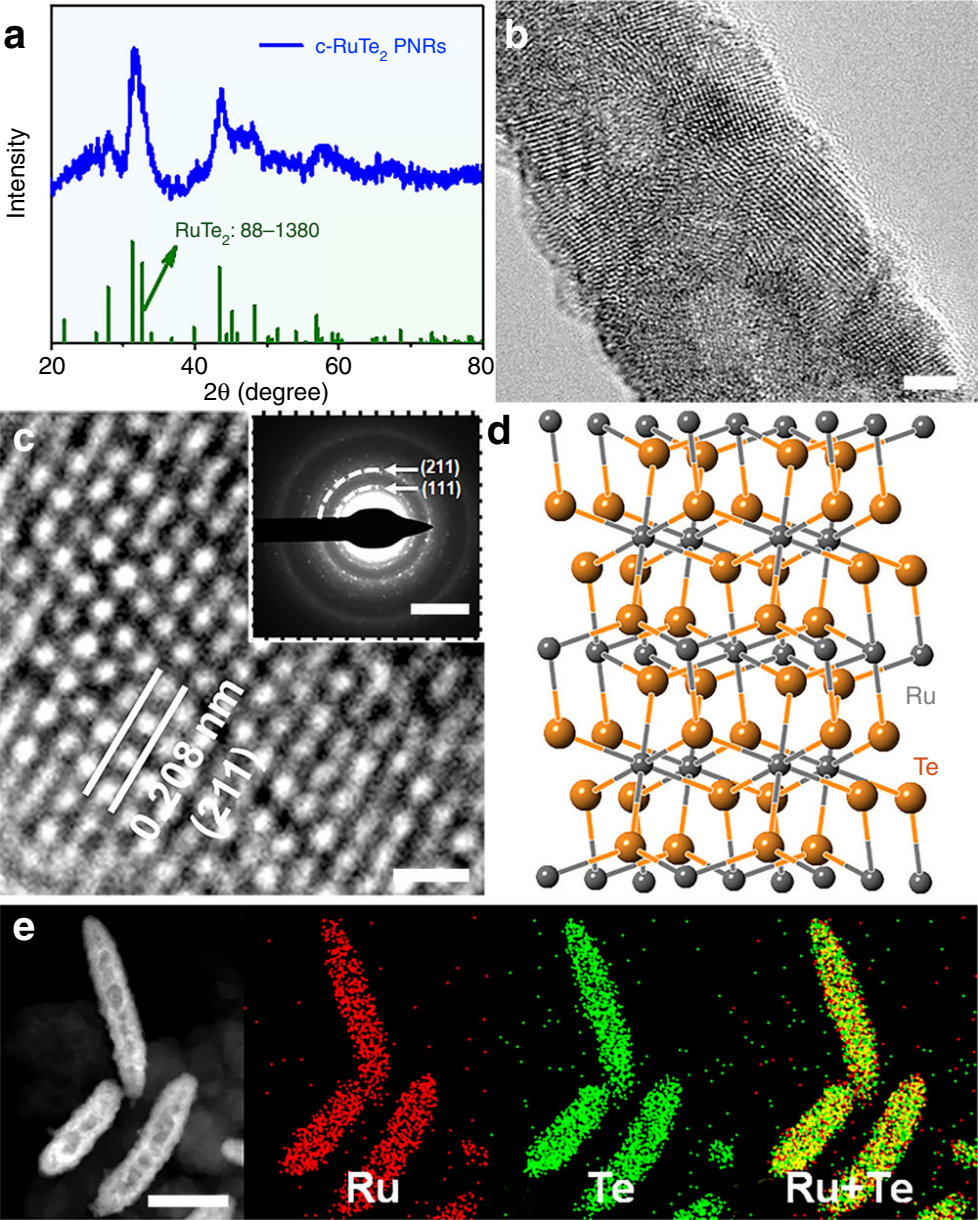
Morphology and structural characterizations. **a** PXRD pattern, **b**, **c** HRTEM image. **d** structural model and **e** STEM-ADF image and EDS elemental mappings of carbon supported c-RuTe_2_ PNRs. Inset in **c** is the SAED pattern of c-RuTe_2_ PNRs. Scale bar, **b** 2 nm; **c** 0.5 nm; (Inset in **c**) 5 1/nm; **e** 40 nm.

The highly crystalline RuTe_2_ PNRs (denoted as c-RuTe_2_ PNRs) were obtained by thermal treating RuTe_2_ PNRs under controlled thermal treatments^34^. PXRD pattern shows two main peaks at 31.29° and 43.37°, corresponding to the (111) and (211) planes of RuTe_2_ (PDF#88-1380), respectively (Fig. 5a). The crystal characteristics and long-range order of c-RuTe_2_ PNRs can also be demonstrated by atomic-resolution HRTEM image (Fig. 5b). Significantly, a large number of regularly arranged atoms can be observed by HRTEM and their interplanar spacing is 0.208 nm, corresponding to the (211) plane of RuTe_2_ (Fig. 5c). SAED pattern also reveals a set of distinct rings composed of diffraction spots (inset in Fig. 5c), being assigned to the (111) and (211) planes. Surprisingly, the morphology and composition of c-RuTe_2_ PNRs are largely maintained, indicating that the controlled thermal treatments only affect the crystallinity but hardly cause morphology and composition changes (Supplementary Fig. 5). The structural model of c-RuTe_2_ PNRs presents a long-range ordered anisotropy with a regular orthorhombic structure (P*nnm*) (Fig. 5d and Supplementary Fig. 6). STEM-EDS elements mappings show that the Ru and Te are uniformly distributed within the whole PNRs (Fig. 5e).

### Evaluation of electrochemical activity

**Fig. 6:**
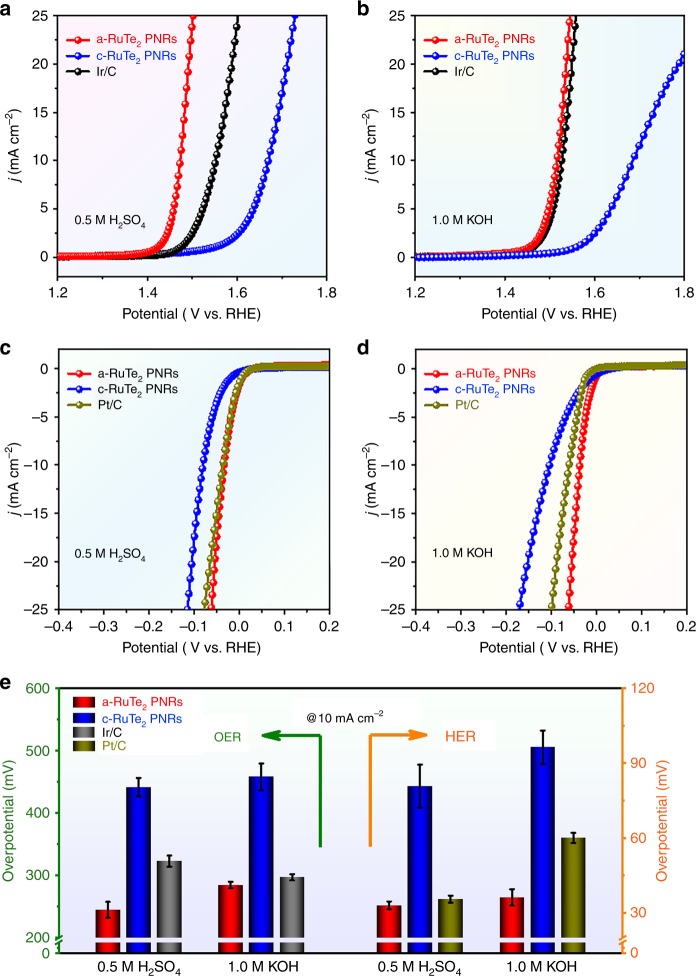
Electrochemical studies of catalysts in different electrolytes. OER polarization curves of a-RuTe_2_ PNRs, c-RuTe_2_ PNRs and commercial Ir/C in **a** 0.5 M H_2_SO_4_ and **b** 1.0 M KOH. HER polarization curves of a-RuTe_2_ PNRs, c-RuTe_2_ PNRs and commercial Pt/C in **c** 0.5 M H_2_SO_4_ and **d** 1.0 M KOH. **e** Histogram of overpotentials at 10 mA cm^−2^ from **a**–**d**.

We performed OER and HER measurements of a-RuTe_2_ PNRs and c-RuTe_2_ PNRs under the universal pH range. Commercial Ir/C and Pt/C were tested under the same conditions for comparison. As expected, the a-RuTe_2_ PNRs show enhanced electrocatalytic performance than that of their crystalline counterparts. In detail, as shown in Fig. 6a, the a-RuTe_2_ PNRs exhibit the best OER activity with a low overpotential of 245 mV at 10 mA cm^−2^ in 0.5 M H_2_SO_4_, which is superior to those of c-RuTe_2_ PNRs (442 mV) and Ir/C (323 mV). Obviously, compared with Ir/C (297 mV) in 1.0 M KOH, only an overpotential of 285 mV is required for a-RuTe_2_ PNRs, while the c-RuTe_2_ PNRs need a higher overpotential of 458 mV under the same conditions, which is highly consistent with the theoretical predictions (Fig. 6b and Supplementary Table 1). Importantly, the a-RuTe_2_ PNRs not only present excellent electrocatalytic performance in the strong acidic and alkaline media but also exhibit outstanding activities in 0.05 M H_2_SO_4_ and 0.1 M KOH (Supplementary Fig. 7). Additionally, the HER activities of various catalysts were investigated. We can see that the HER performance of a-RuTe_2_ PNRs is significantly better than that of c-RuTe_2_ PNRs, and even exceed that of Pt/C as the applied voltage increasing (Fig. 6c and Supplementary Table 2). Although all catalysts show similar catalytic activity in 1.0 M KOH at the beginning, the overpotential of a-RuTe_2_ PNRs is markedly lower than those of c-RuTe_2_ PNRs and Pt/C with increasing voltage (Fig. 6d). Similarly, robust HER activity in 0.05 M H_2_SO_4_ and 0.1 M KOH can also be observed for a-RuTe_2_ PNRs (Supplementary Fig. 8). To further analyze the electrocatalytic activity of various catalysts, we summarize the overpotential at a current density of 10 mA cm^−2^ in different electrolytes (Fig. 6e). Evidently, the OER and HER properties of a-RuTe_2_ PNRs are superior to those of c-RuTe_2_ PNRs, Ir/C and Pt/C, especially in the acidic environment. Tafel slopes of various catalysts were also summarized in Supplementary Figs. 9, 10, in which the a-RuTe_2_ PNRs show the smallest Tafel slope in different pH ranges, representing the fastest reaction kinetics. To evaluate the number of active sites, the copper underpotential deposition (Cu UPD) method was performed^35,36^. Compared with c-RuTe_2_ PNRs, an increase in the number of active sites can be observed for a-RuTe_2_ PNRs, and thus exhibiting improved catalytic activity (Supplementary Fig. 11). Cyclic voltammetry curves were performed in Supplementary Fig. 12, no redox peaks appeared for a-RuTe_2_ PNRs during OER process, demonstrating that their self-oxidation is negligible. Faraday efficiency (FE) of OER and HER are more than 95%, indicating that the high currents are almost entirely originated from the process of water splitting (Supplementary Fig. 13). In addition, the HER and OER polarization curves of a-RuTe_2_ without iR correction are also performed as reference (Supplementary Fig. 14), where the a-RuTe_2_ PNRs exhibit excellent OER and HER activities under different conditions.

**Fig. 7:**
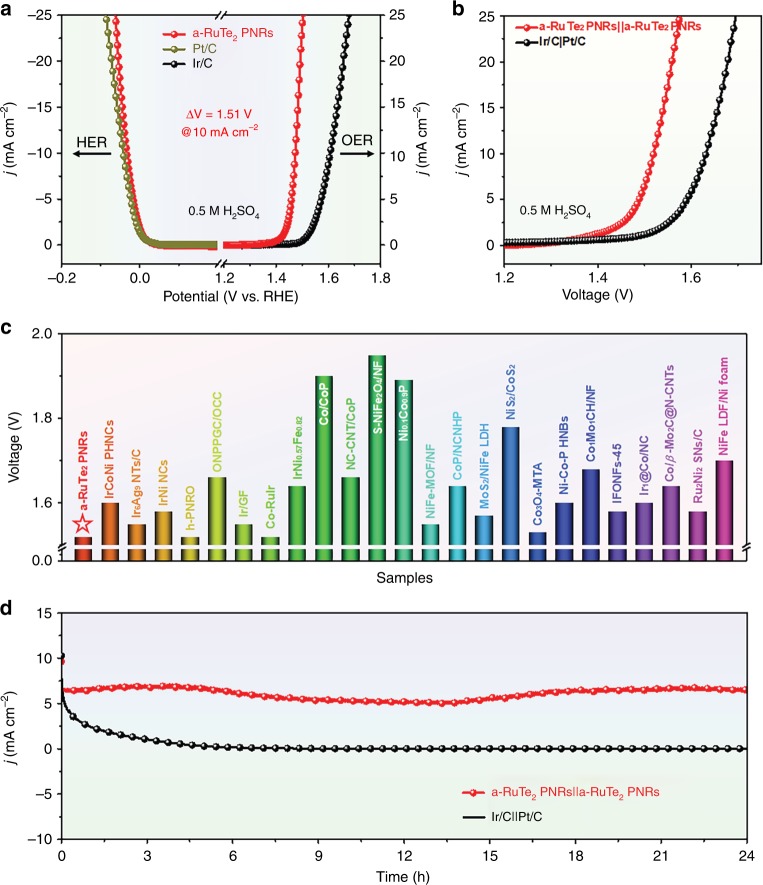
Overall water splitting studies of catalysts in acidic media. **a** Polarization curves of a-RuTe_2_ PNRs, commercial Pt/C and Ir/C in 0.5 M H_2_SO_4_ for HER and OER. **b** Polarization curves of a-RuTe_2_ PNRs||a-RuTe_2_ PNRs and Ir/C||Pt/C in 0.5 M H_2_SO_4_ for water splitting. **c** Comparison of the overpotential of a-RuTe_2_ PNRs and recently reported overall water splitting electrocatalysts at 10 mA cm^−2^. **d** Time-dependent current density curves of a-RuTe_2_ PNRs||a-RuTe_2_ PNRs and Ir/C||Pt/C for water splitting in 0.5 M H_2_SO_4_.

Considering the superior HER and OER activities of a-RuTe_2_ PNRs in acidic condition, a two-electrode device for acidic overall water splitting was constructed. Figure 7a displays the polarization curves of a-RuTe_2_ PNRs, Pt/C and Ir/C in 0.5 M H_2_SO_4_. At left, the a-RuTe_2_ PNRs exhibit a slightly improved HER activity compared with Pt/C. However, at right, the OER activity of a-RuTe_2_ PNRs is significantly better than that of Ir/C. The voltage difference (ΔV) between HER and OER at the current density of 10 mA cm^−2^ is 1.51 V. When a-RuTe_2_ PNRs were employed as both anode and cathode catalysts in 0.5 M H_2_SO_4_, only a cell voltage of 1.52 V is needed to achieve 10 mA cm^−2^, which is much better than that of Ir/C||Pt/C (Fig. 7b). Most importantly, the cell voltage of a-RuTe_2_ PNRs||a-RuTe_2_ PNRs for water splitting at 10 mA cm^−2^ even surpasses most reported electrocatalysts (Fig. 7c and Supplementary Table 3). The chronoamperometry curve was then carried out to evaluate the long-term stability of a-RuTe_2_ PNRs||a-RuTe_2_ PNRs (Fig. 7d). Compared with Ir/C||Pt/C, the a-RuTe_2_ PNRs||a-RuTe_2_ PNRs exhibit improved stability in acidic condition (24 h). Detailed characterizations were carried out to further understand the enhanced activity and stability of a-RuTe_2_ PNRs for water splitting in acidic conditions (Supplementary Fig. 15, 16). We can see that the porous morphology and bullet-like outline were largely maintained while Ir/C and Pt/C were severely agglomerated (Supplementary Figs. 17, 18). The amorphous feature of a-RuTe_2_ PNRs can also be largely maintained. Additionally, the a-RuTe_2_ PNRs can maintain excellent chemical stability even under 5.0 M H_2_SO_4_ at 60 °C for 1 h (Supplementary Fig. 19). Therefore, the a-RuTe_2_ PNRs not only exhibit excellent chemical corrosion resistance but also present superior electrochemical stability.

### Mechanistic investigations

**Fig. 8:**
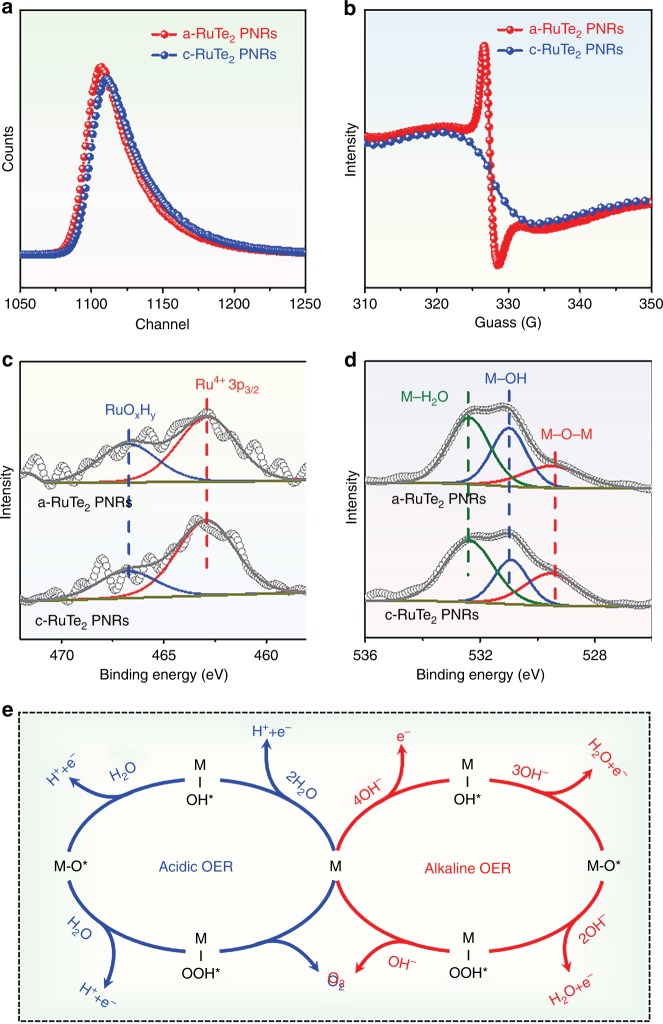
Mechanism investigations. **a** Positron lifetime spectra, **b** ESR spectra, **c** Ru 3p, and **d** O 1s XPS spectra of a-RuTe_2_ PNRs and c-RuTe_2_ PNRs. **e** Schematic illustration of the OER process under acidic and alkaline conditions.

To further explore the reasons for the significant activity difference of a-RuTe_2_ PNRs and c-RuTe_2_ PNRs, electrochemically active surface area (ECSA) was calculated (Supplementary Fig. 20 and Supplementary Table 4). Apparently, an improved ECSA that represents more exposed active sites can be observed for a-RuTe_2_ PNRs, which may be caused by the distortions of Ru-Te bond that derived from the high density of defects in amorphous materials. This finding will also verify the previous theoretical investigations on the local electronic structures. Positron annihilation spectroscopy (PAS) and corresponding lifetime parameters were then performed (Fig. 8a and Supplementary Table 5). We can see that the a-RuTe_2_ PNRs exhibit a significantly prolonged lifetime (τ_1_ and τ_2_) than their crystalline counterparts, mainly due to high density of defects that changed the electron density. To prove this conjecture, electron spin resonance (ESR) measurement was also carried to probe the unpaired electrons that generated by defects (Fig. 8b), in which a pair of sharp peaks can be clearly observed for a-RuTe_2_ PNRs when compared with c-RuTe_2_ PNRs, supporting the presence of a high density of defects in the a-RuTe_2_ PNRs. In fact, the presence of defective sites can not only affect the electronic distribution caused by the distortion of Ru-Te bond but also combine with oxygen atoms to form RuO_x_H_y_ species which are conducive the reaction process^37^. Therefore, X-ray photoelectron spectroscopy (XPS) was employed to measure the chemical composition and electronic configuration. As shown in Fig. 8c, compared with c-RuTe_2_ PNRs, a significantly enhanced peak area of RuO_x_H_y_ species can be observed for a-RuTe_2_ PNRs, indicating that the presence of defects was filled with oxygen and then to form OH species. The increased OH species in a-RuTe_2_ PNRs can also be demonstrated by O 1s XPS (Fig. 8d), in which the relative peak area of M-OH species increase, whereas the M-O-M species decrease. In view of the experimental results and theoretical analysis, possible OER mechanisms under pH-universal conditions have been depicted in Fig. 8e. As illustrated, for acidic OER process, water molecule preferentially splitting on the catalyst's surface and then gradually dehydrogenate to form M-OOH* intermediate. Finally, O_2_ is successfully released directly from the surface and the active site continues to proceed with the catalytic reaction. Under the alkaline condition, the dissociated OH^−^ is adsorbed on the catalyst's surface and then form M-OH* intermediate. Subsequently, the M-OH* combines with OH^−^ to release water molecule and then becomes M-O*. The metastable M-O* intermediate will soon combine with OH^−^ to generate M-OOH* and eventually releases O_2_.

## Discussion

In summary, theoretical calculations have supplied an insightful understanding of the intrinsic high electroactivity that directionally guide the experimental synthesis of the a-RuTe_2_ PNRs as the efficient water splitting electrocatalysts. DFT calculations reveal that the local flexible Te-bonding configurations are yielded from a strong p-d transfer induced p-π sensitivity enhancement, which renders the stabilization of distortion-strain as well as elevates electronic activities near the Fermi level through an effective annihilation of the crystal-field-splitting effect of Ru-sites. Within this trend, the local distorted Ru-Te lattice increases the homogeneity for efficient inter-d-orbital electron-transfer ability among Ru sites. Therefore, the short-range disorder promotes the electron-lattice coupling effect but also boosts OER catalysis within pH-universal conditions. As a result, the a-RuTe_2_ PNRs exhibit superior HER and OER activities than those of their crystalline counterparts. More importantly, a relatively low cell potential of 1.52 V has been achieved for reaching the current density of 10 mA cm^−2^ in water splitting, representing much enhanced activity under acidic conditions. Detailed investigations show that the generation of distorted Ru-Te bonds is attributed to the extensive defects in the amorphous structure. These defects will be substituted by oxygen atoms to form RuO_x_H_y_ species that will promote the catalytic activity. Our work provides a feasible strategy in amorphous catalysts design and investigation that offers valuable insight to the development of a new generation of catalysts, which will broaden the horizon of future electrocatalyst studies in energy applications.

## Materials and methods

### Chemicals

Potassium tellurite (K_2_TeO_3_, 99.5%) was purchased from Aladdin-reagent Inc. Hexaammineruthenium (III) chloride (Cl_3_H_18_N_6_Ru, Ru 32.1%) was purchased from Alfa Aesar. Poly (vinylpyrrolidone) (PVP, average M.W. 58000, K15-19) was purchased from J&K Scientific Ltd. Ammonia solution (NH_3_·H_2_O, AR), hydrazine hydrate aqueous solution (N_2_H_4_·H_2_O, AR) and isopropanol (IPA, AR) were purchased from Sinopharm Chemical Reagent Co., Ltd. Argon (Ar, 99.999%) was purchased from WuGang Gas Co., Ltd. (Shanghai, China). Pt/C (20 wt% Pt on Vulcan black) was purchased from Shanghai Hesen Electric Co., Ltd. Ir/C (20 wt% Ir on Vulcan black) was from Premetek Co., Ltd.

### Synthesis of RuTe_2_ PNRs

3.6 mg Cl_3_H_18_N_6_Ru (11.6 µmol), 6.0 mg K_2_TeO_3_ (23.6 µmol) and 65.0 mg PVP (1.1 µmol) were dissolved in 2 mL H_2_O. After a few minutes of sonication, 2.0 mL NH_3_·H_2_O and 1.0 mL N_2_H_4_·H_2_O were quickly injected into the above mixture. The mixture solution was then transferred into Teflon-sealed autoclave and maintained at 180 °C for 3 h. The RuTe_2_ PNRs were obtained by washed several times with ethanol/acetone solution.

### Synthesis of a-RuTe_2_ PNRs and c-RuTe_2_ PNRs

RuTe_2_ PNRs were deposited on VC-X72 carbon (Ru loading of 20 wt%, determined by ICP-AES) in ethanol solution by sonicating for 30 min. The resulting products were separated by centrifugation and washed several times using ethanol/acetone solution. The products were annealed at 250 °C in air for 5 h to yield a-RuTe_2_ PNRs. The c-RuTe_2_ PNRs were obtained by annealing treatment at 250 °C in Ar for 5 h and then at 250 °C in air for 1 h.

### Characterization

Low-magnification transmission electron microscopy (TEM) was performed on a HITACHI HT7700 at 120 kV. High-angle annular dark-field scanning transmission electron microscopy (HAADF-STEM) and HRTEM was recorded on a FEI Talos F200X S/TEM with a field-emission gun at 200 kV. ESR spectra were collected on JEOL JES-X320. XPS was performed on SSI S-Probe XPS Spectrometer. Powder X-ray diffraction (PXRD) patterns were collected on X’Pert-Pro MPD diffractometer (Netherlands PANalytical) with a Cu Kα X-ray source (*λ* = 1.540598 Å).

### Electrochemical measurements

Electrochemical measurements were performed on CHI660 workstation (Chenhua, Shanghai) by using the three-electrode system. Graphite rod and saturated calomel electrode were used as counter and reference electrode, respectively. To prepare the catalyst ink, 2 mg catalysts were added into a mixture solution including 800 µL IPA, 200 µL H_2_O and 5 µL Nafion. After 30 min sonication, 20 µL catalyst ink was deposited on glassy carbon electrode (diameter 5 mm, area: 0.196 cm^2^) as a working electrode. Polarization curves were then performed in a broad pH range, including 0.5 M H_2_SO_4_, 0.05 M H_2_SO_4_, 0.1 M KOH and 1.0 M KOH, after a continuous cyclic voltammetry. All polarization curves in this study are the average of the stable polarization curves scanned in three experiments. The solution resistance (Rs) is ~6 Ω in 0.5 M H_2_SO_4_, ~39 Ω in 0.05 M H_2_SO_4_, ~36 Ω in 0.1 M KOH and ~5 Ω in 1 M KOH. The Tafel slopes were derived from polarization curves and 95% iR compensation in all the solutions. Long-term stability for water splitting was tested by using a two-electrode system. The underpotential deposition (UPD) method was used to qualify active sites. The number of active sites can be calculated with the equation: *n* = Q_Cu_/2F, where Q is the UPD Cu stripping charge (Q_Cu_, Cu_upd_ → Cu^2+^ + 2e^−^) and F is the Faraday constant. The Faraday efficiency (FE) measurements were conducted on an H-cell reactor where each chamber of ~60 mL was filled with 30 mL of 0.5 M H_2_SO_4_ solution and the two chambers were separated by an anion exchange membrane (Nafion 117). The Ar (30 sccm) was applied throughout the HER and OER measurements. Chronoamperometry measurements were carried out at the voltage that the current density reached 10 mA cm^−2^. The reactor was directly connected to the gas chromatograph (GC Agilent 7890B). The FE of a product was calculated as follows: FE = *e*F × *n*/*Q*, where e is the number of electrons transferred of the product, Q is the total charge in HER and OER process, n is the number of moles of the product and F is the Faraday constant.

### Computational details

Rotationally invariant DFT+U calculations within CASTEP code has been performed^38,39^. The algorithm of Broyden-Fletcher-Goldfarb-Shannon (BFGS) has chosen for all related ground state geometry optimization, especially for the interfacial relaxation. The cutoff energy of plane-wave basis sets for total energy and valence electronic states calculations has been set to 750 eV. The PBE exchange-correlation functional is selected for DFT+U calculations. The applied U values in this work are 2.38 and 0.36 eV for Ru and Te, respectively. To improve the convergence quality of the transition metal compound system, the ensemble DFT (EDFT) method of Marzari et al. is used during the electronic-minimization process^40^.

To approach a realistic local short-range ordered structure, a fixed volume NVT ensemble has been used for ab-initio molecular dynamics (AIMD) to conduct an analogue anneal-to-quench process from 1600 to 300 K. All of these AIMD simulations have been performed onto an expanded supercell of crystalline RuTe_2_ lattice with a density of 6.036 g/cm^3^ (8.303 g/cm^3^ for crystalline). The original crystalline RuTe_2_ unit-cell lattice has been imported from the database with group symmetry of PNNM and experimental lattice parameters (*a* = 5.38 Å, *b* = 6.49 Å, and *c* = 4.08 Å). The geometry optimization is also applied to the randomly selective trajectories of the MD process. The weighted average coordination (CN) of Ru is lowered (CN = 6 for crystalline RuTe_2_) staying between 4 (tetrahedral) and 6 (octahedral), while the Te sites are more flexible ranged from CN = 2 to CN = 6 containing Te-Te bonds. Considering the DFT computational cost, the Monkhost-Pack reciprocal space integration was performed using Gamma-center-off special k-points with a mesh of 2 × 2 × 2, which was guided by the initial convergence test^41^. With these settings, the overall total energy for each step is converged to less than 5.0 × 10^−7^ eV per atom. The Hellmann-Feynman forces on the atom were converged to less than 0.001 eV/Å.

The Ru, Te, O, and H norm-conserving pseudopotentials are generated using the OPIUM code in the Kleinman-Bylander projector form, and the non-linear partial core correction and a scalar relativistic averaging scheme are used to treat the mixed valence Co spin-orbital coupling effect^42–44^. We chose the projector-based (4*d*, 5*s*, 5*p*), (5*s*, 5*p*), (2*s*, 2*p*), and (1*s*) states to reflect the valence states of Ru, Te, O, and H atoms, respectively. The RRKJ method is chosen for the optimization of the pseudopotentials^45^.

To achieve the mass and electron conservation, the key adsorbates and co-reactants are both considered in the calculation of the energetic diagram for the water splitting reactions. For each intermediate, we have fully considered the possible diffusions on the surface to locate the most stable adsorption site and corresponding coordination number (CN) environment in the complicated amorphous structure modelling.

For the acidic OER, the acidic OER reactions are as below.

1 $${\mathrm{2H}}_{\mathrm{2}}{\mathrm{O}}^ \ast \to {\mathrm{OH}}^ \ast + {\mathrm{H}}_{\mathrm{2}}{\mathrm{O}} + \left( {{\mathrm{H}}^{\mathrm{ + }} + {\mathrm{e}}^{\mathrm{ - }}} \right)$$

2 $${\mathrm{OH}}^ \ast + {\mathrm{H}}_{\mathrm{2}}{\mathrm{O}} + \left( {{\mathrm{H}}^{\mathrm{ + }}{\mathrm{ + e}}^{\mathrm{ - }}} \right) \to {\mathrm{O}}^ \ast + {\mathrm{H}}_{\mathrm{2}}{\mathrm{O}} + \left( {{\mathrm{2H}}^{\mathrm{ + }}{\mathrm{ + 2e}}^{\mathrm{ - }}} \right)$$

3 $${\mathrm{O}}^ \ast + {\mathrm{H}}_{\mathrm{2}}{\mathrm{O}} + \left( {{\mathrm{2H}}^{\mathrm{ + }}{\mathrm{ + 2e}}^{\mathrm{ - }}} \right) \to {\mathrm{OOH}}^ \ast + \left( {{\mathrm{3H}}^{\mathrm{ + }}{\mathrm{ + 3e}}^{\mathrm{ - }}} \right)$$

4 $${\mathrm{OOH}}^ \ast + \left( {{\mathrm{3H}}^{\mathrm{ + }}{\mathrm{ + 3e}}^{\mathrm{ - }}} \right) \to {\mathrm{O}}_{\mathrm{2}}\left( {\mathrm{g}} \right) + \left( {{\mathrm{4H}}^{\mathrm{ + }}{\mathrm{ + 4e}}^{\mathrm{ - }}} \right)$$

On the contrary, the OER process in alkaline medium renders reactions are as below.

5 $${\mathrm{4OH}}^{\mathrm{ - }} \to {\mathrm{OH}}^ \ast + {\mathrm{3OH}}^{\mathrm{ - }} + {\mathrm{e}}^{\mathrm{ - }}$$

6 $${\mathrm{OH}}^ \ast + {\mathrm{3OH}}^{\mathrm{ - }} + {\mathrm{e}}^{\mathrm{ - }} \to {\mathrm{O}}^ \ast + {\mathrm{2OH}}^{\mathrm{ - }} + {\mathrm{H}}_{\mathrm{2}}{\mathrm{O}} + {\mathrm{2e}}^{\mathrm{ - }}$$

7 $${\mathrm{O}}^ \ast + {\mathrm{2OH}}^{\mathrm{ - }} + {\mathrm{H}}_{\mathrm{2}}{\mathrm{O}} + {\mathrm{2e}}^{\mathrm{ - }} \to ^ \ast {\mathrm{OOH}} + {\mathrm{OH}}^{\mathrm{ - }} + {\mathrm{2H}}_{\mathrm{2}}{\mathrm{O}} + {\mathrm{3e}}^{\mathrm{ - }}$$

8 $$\ast {\mathrm{OOH}} + {\mathrm{OH}}^{\mathrm{ - }} + {\mathrm{2H}}_{\mathrm{2}}{\mathrm{O}} + {\mathrm{3e}}^{\mathrm{ - }} \to {\mathrm{O}}_{\mathrm{2}} + {\mathrm{2H}}_{\mathrm{2}}{\mathrm{O}} + {\mathrm{4e}}^{\mathrm{ - }}$$

## Supplementary information

Supplementary Information

## Data Availability

The data that support the findings of this study are available from the corresponding author upon request.
