# Supplementary material for: Amorphization activated ruthenium-tellurium nanorods for efficient water splitting
Source: Nat Commun. 2019 Dec 12;10:5692. doi: 10.1038/s41467-019-13519-1 (PMC6908605; doi:10.1038/s41467-019-13519-1)
Supplement: Supplementary file 1 — Supplementary Information [file 41467_2019_13519_MOESM1_ESM.pdf]

Supporting Information for

Amorphization activated ruthenium-tellurium nanorods for efficient water-splitting

Wang et al.

Supplementary Note 1

Amorphous Structure Activated Electroactivity Enhancement

The classical density functional theory (DFT) proposed that the ground-state electronic energy is a function of the density as Eq. (1).

$$E[\rho] = \int \rho(1)v(1)d\tau_1 + F[\rho] \qquad \text{Supplementary Equations (1)}$$

In the above equation, the v is the external one-particle potential and F[ρ] is the sum of electronic kinetic energy and electron repulsion energy. To further overcome the onsite semi-core orbital self-energy effect, we introduce the Lagrange multiplier within the DFT+U [1-5], in which the ground-state electronic energy is derived from $E[\rho]$ to $E[N]$. Since the stationary principle for the density and energy is written as $\delta\{E_v[\rho^{'}]\text{-}\mu N[\rho^{'}]\} = 0$ (μ is the Lagrange multiplier), the calculation of variations follows the equation below. The associated value of μ is characteristic of the system of interest and is commonly called the chemical potential of the system.

$$\mu = \left(\frac{\delta E}{\delta N}\right)_v \qquad \text{Supplementary Equations (2)}$$

In addition, they also prove the electronegativity as a constant throughout an atom or molecule, and from orbital to orbital within an atom or molecule. The valence-state electronegativity differences drive charge transfers towards the formation of the molecule.

Furthermore, we apply the Tyler expansion to derive $E[N]$ towards the following expressions.

$$E(N) = aN + bN^2 + c(N^3) + \cdots + (o) \quad \text{Supplementary Equations (3)}$$

Thus, the electronegativity μ in Eq. (2) will be changed to the expressions as below.

$$\mu = \frac{\delta E[N]}{\delta N} = a + 2bN + \cdots + (o) \qquad \text{Supplementary Equations (4)}$$

As the deviation of electronegativity, the electroactivity expression of a crystalline will become as below.

$$\left[\frac{\delta E^2[N]}{\delta N^2}\right]_{xtal} = 2b + (o) \qquad \text{Supplementary Equations (5)}$$

Therefore, the 2b constant determines the electroactivity of materials, which is highly correlated to the electron transfer probability. The amorphous structure also correlates to a different constant of electroactivity as Eq. (6).

$$\left[\frac{\delta E^2[N]}{\delta N^2}\right]_{am} = 2b' + (o') \qquad \text{Supplementary Equations (6)}$$

Due to the much larger electron transfer probability in the amorphous structure, $2b'$ of the amorphous structure is much larger than the $2b$ of the crystalline, supporting the enhanced electroactivity.

$$2b^{'} \gg 2b \qquad \text{Supplementary Equations (7)}$$

In a given solid crystal system, the probability of electron transfer is given as below,

$$W_{i\to f}(E) \propto \frac{2\pi}{\hbar} |\langle M\rangle|^2 \delta\,(E_f\text{-}E_i\text{-}E) \qquad \text{Supplementary Equations (8)}$$

In this expression, *i* denotes the initial state and f the final state; $|\langle M\rangle|$ is the matrix elements of electrical dipole-dipole oscillator strength of transitions introduced by the Fermi-Golden Rule. By applying the integral by band theory, Eq. (8) can be reformed as below.

$$W_{i\to f}(E) \propto \frac{2\pi}{\hbar} |\langle M\rangle|^2 \int N_{VB}(E')N_{CB}(E\text{-}E')dE' \qquad \text{Supplementary Equations (9)}$$

The larger matrix element indicates the higher electron transfer strength, which is limited in the crystalline. In particular, the corresponding Coulombic barriers of the inter-orbital electron transfer and site-to-site electron migration of semiconductors are much stronger than in amorphous structures. Thus, our additional calculations with the detailed explanation demonstrate the distinct electron transfer paths of crystalline and amorphous structures, in which the much larger electron transfer probability ($2b_{xtal}$ << $2b_{am}$) in amorphous structures supports the enhanced electroactivity in amorphous $RuTe_2$.

Supplementary Figures

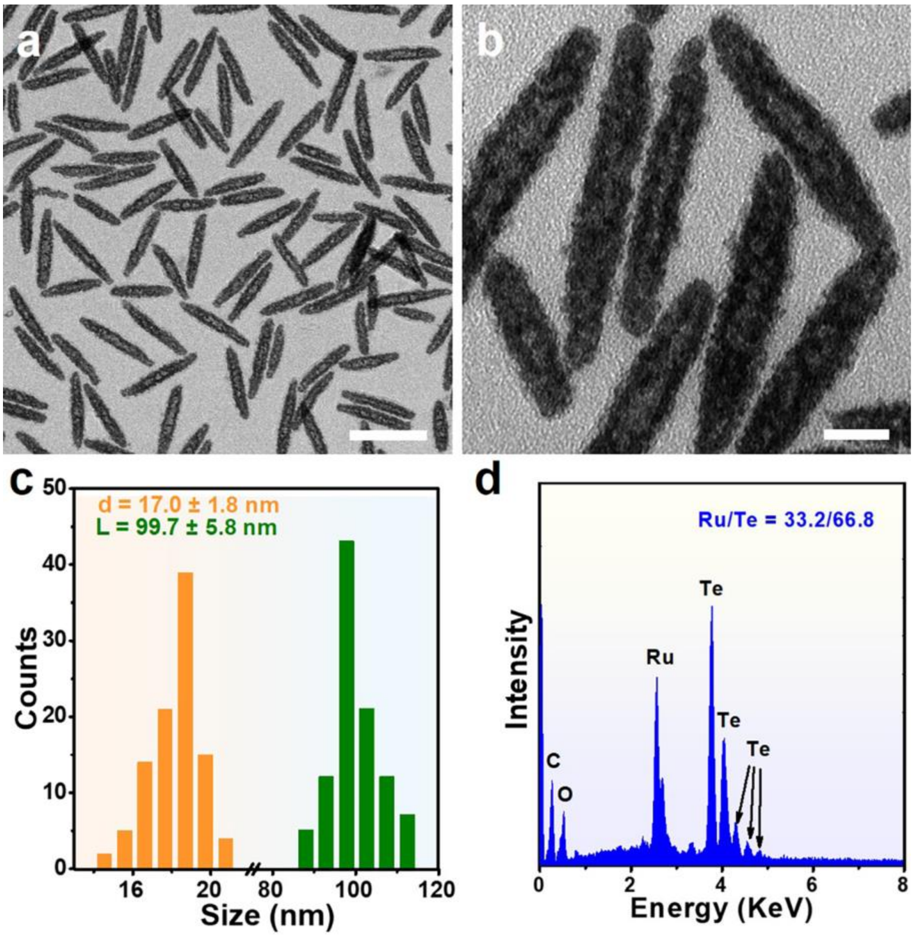

Supplementary Figure 1. (a, b) TEM images, (c) diameter and length histograms and (d) SEM-EDS spectrum of $RuTe_2$ PNRs. Scale bars: (a) 100 nm; (b) 20 nm.

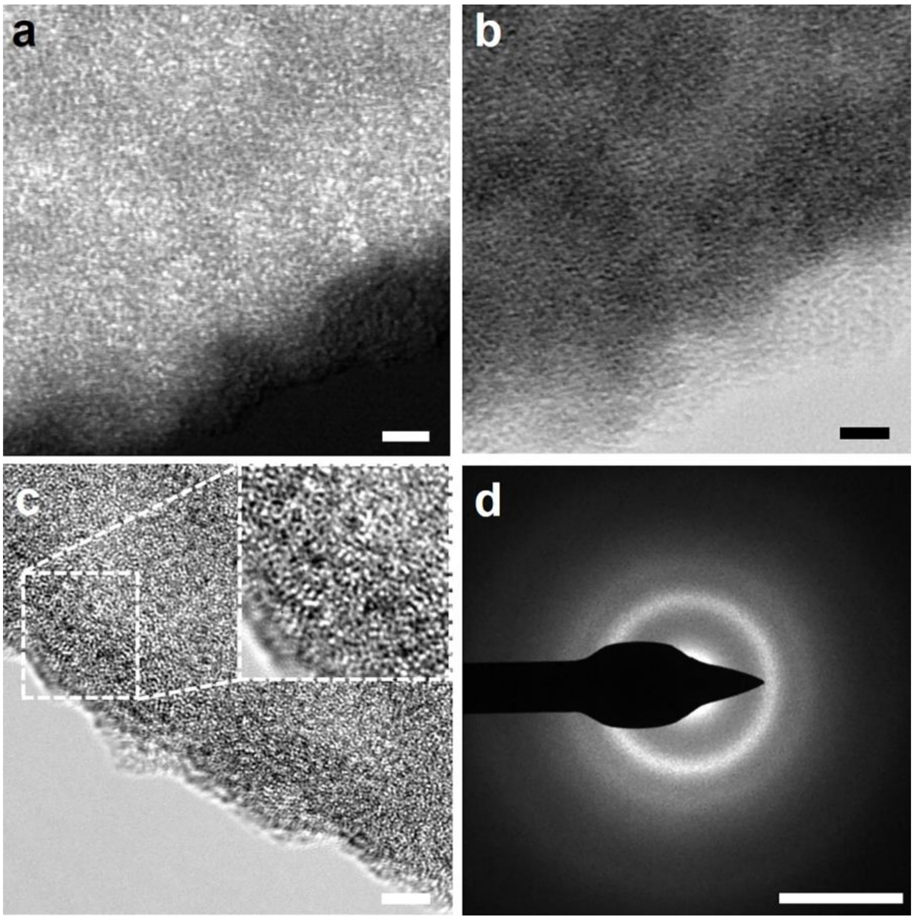

Supplementary Figure 2. Atomic-resolution STEM images in (a) annular dark field and (b) bright-field, (c) HRTEM image and (d) SAED pattern of $RuTe_2$ PNRs. Scale bars: (a-c) 2 nm; (d) 5 1/nm.

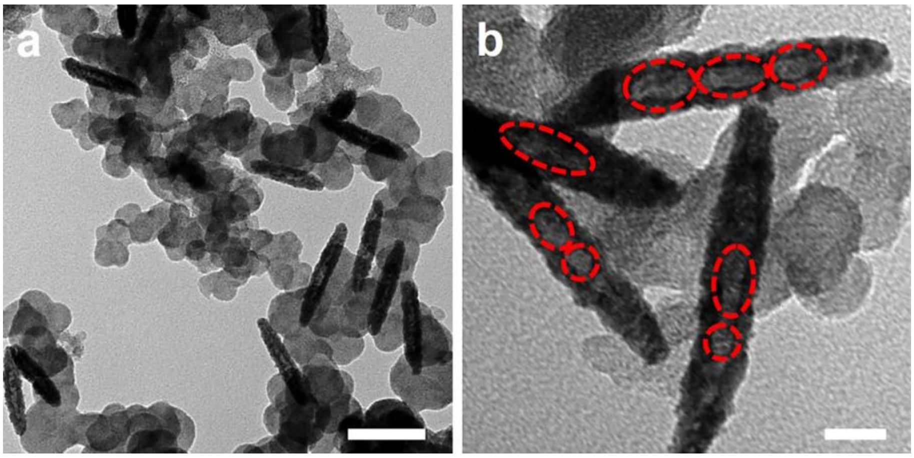

Supplementary Figure 3. TEM images of carbon supported $RuTe_2$ PNRs. Red dashed circles highlight the presence of porous. Scale bars: (a) 100 nm; (b) 20 nm.

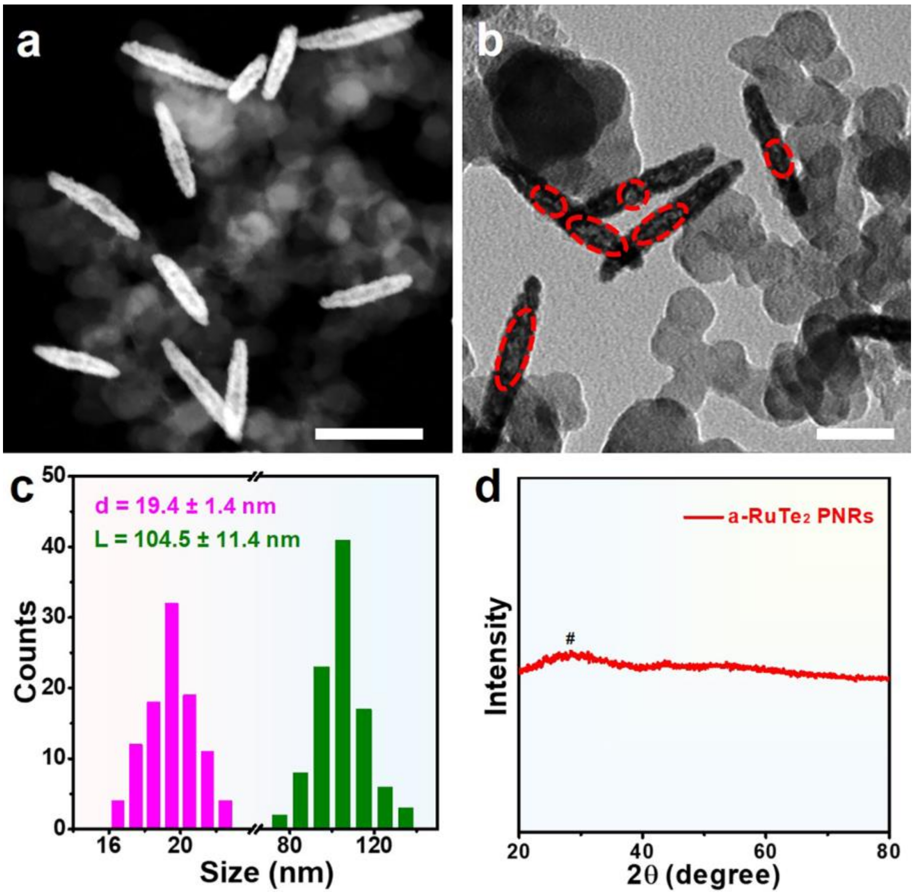

Supplementary Figure 4. (a) HAADF-STEM image, (b) TEM image, and (c) diameter and length histograms and (d) PXRD pattern of carbon supported a-$RuTe_2$ PNRs. Red dashed circles in (b) highlight the presence of porous. Scale bars: (a) 100 nm; (b) 50 nm.

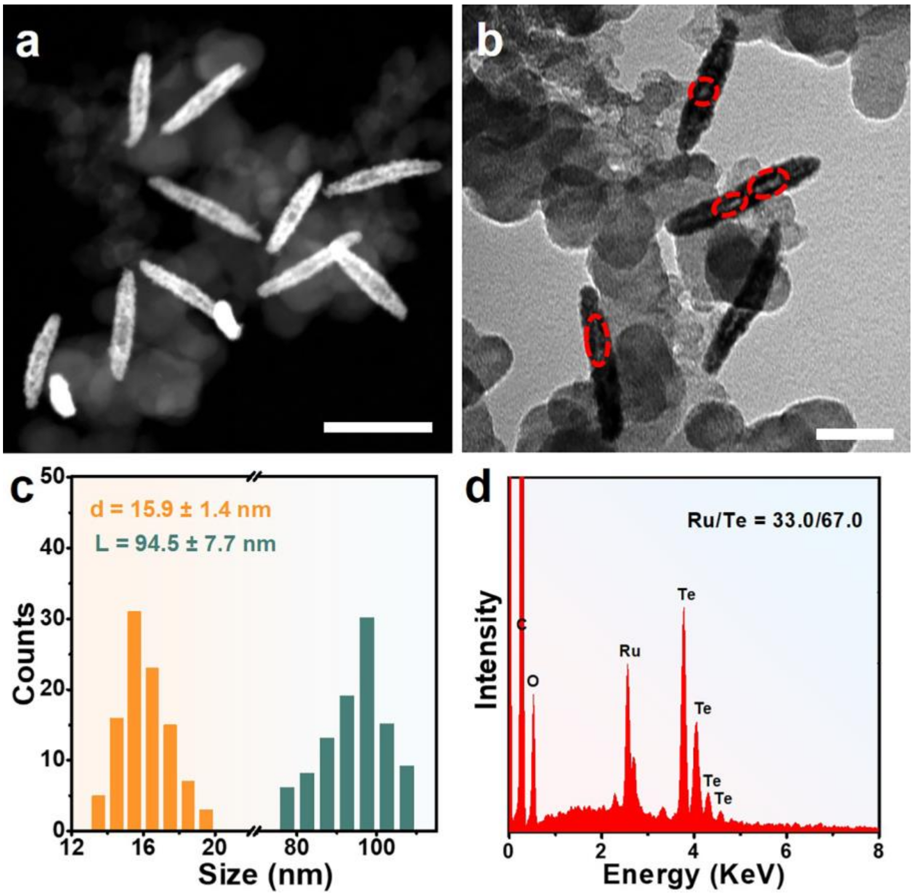

Supplementary Figure 5. (a) HAADF-STEM image, (b) TEM image, and (c) diameter and length histograms and (d) SEM-EDS spectrum of carbon supported c-$RuTe_2$ PNRs. Red dashed circles in (b) highlight the presence of porous. Scale bars: (a) 100 nm; (b) 50 nm.

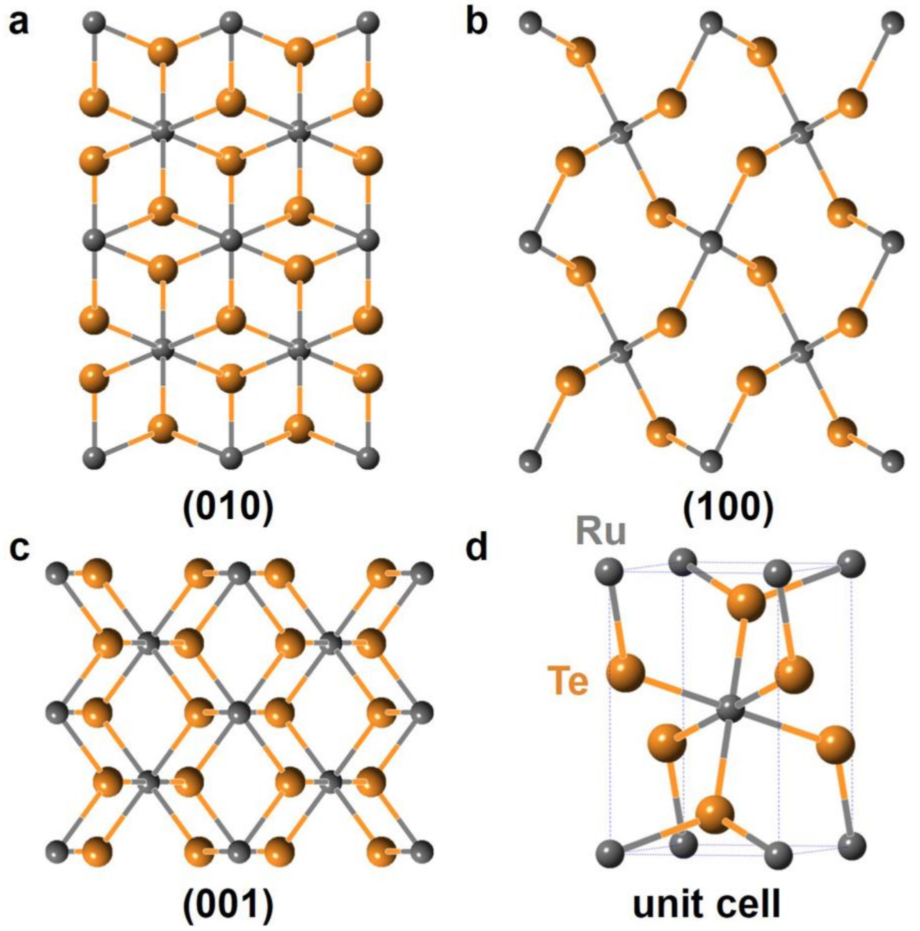

Supplementary Figure 6. Structural models of c-$RuTe_2$ PNRs from (a-c) different view and (d) unit cell.

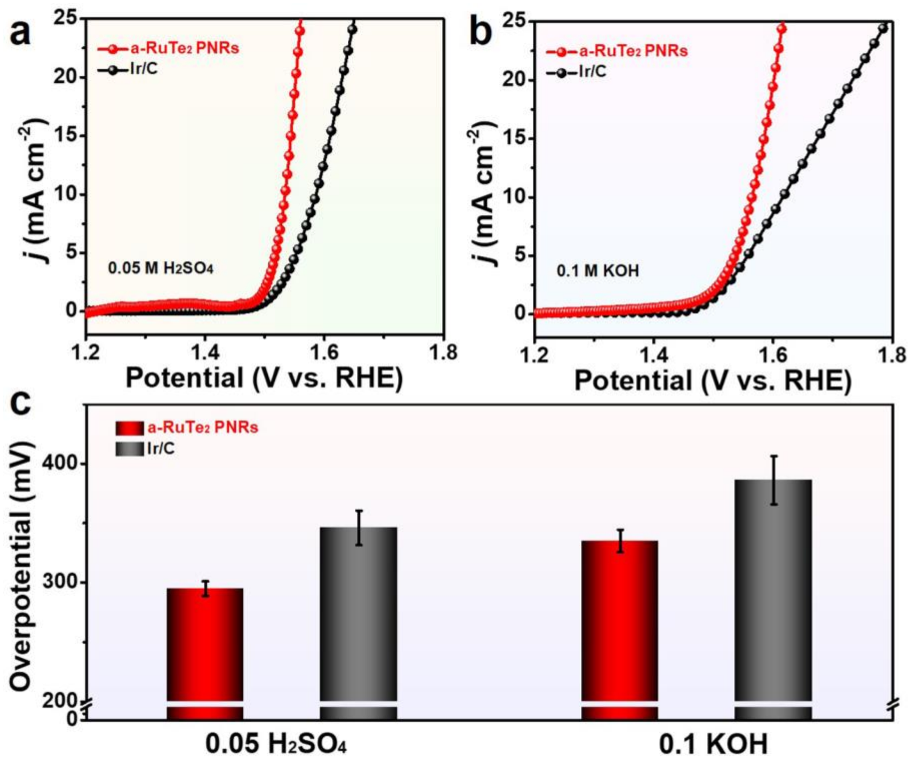

Supplementary Figure 7. OER polarization curves of a-$RuTe_2$ PNRs and Ir/C in (a) 0.05 M H_2SO_4 and (b) 0.1 M KOH. (c) Histogram of overpotentials at 10 mA cm^{-2} from (a, b).

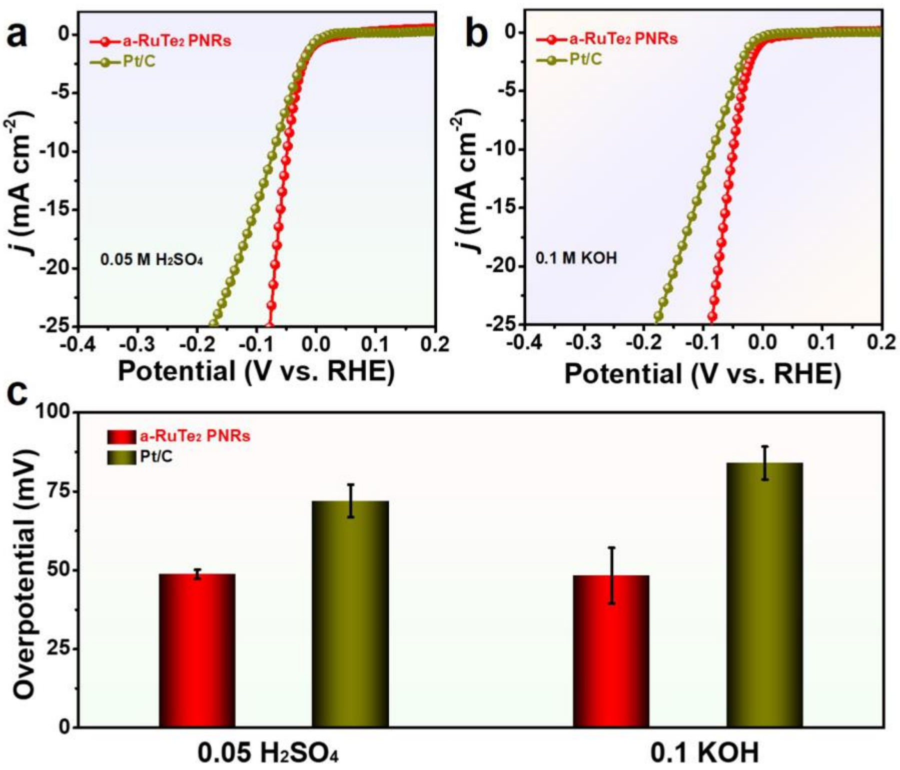

Supplementary Figure 8. HER polarization curves of a-$RuTe_2$ PNRs and Pt/C in (a) 0.05 M H_2SO_4 and (b) 0.1 M KOH. (c) Histogram of overpotentials at 10 mA cm^{-2} from (a, b).

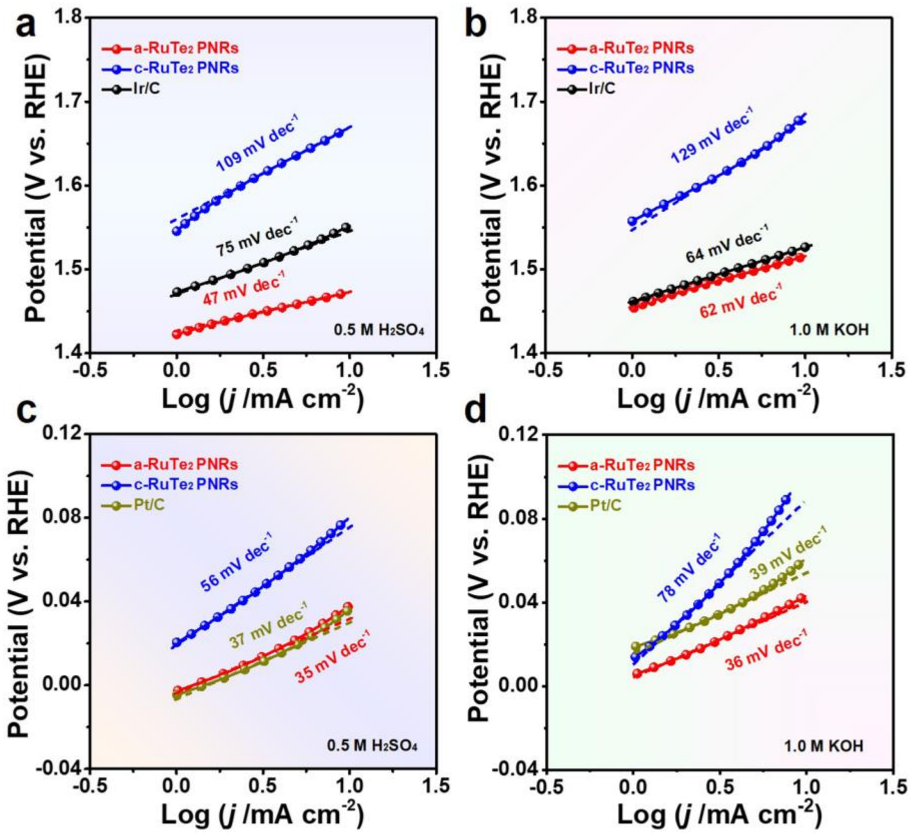

Supplementary Figure 9. OER Tafel slope of a-$RuTe_2$ PNRs, c-$RuTe_2$ PNRs and Ir/C derived from Figure 6a, b in (a) 0.5 M H_2SO_4 and (b) 1.0 M KOH. HER Tafel slope of a-$RuTe_2$ PNRs, c-$RuTe_2$ PNRs and Pt/C derived from Figure 6c, d in (c) 0.5 M H_2SO_4 and (d) 1.0 M KOH.

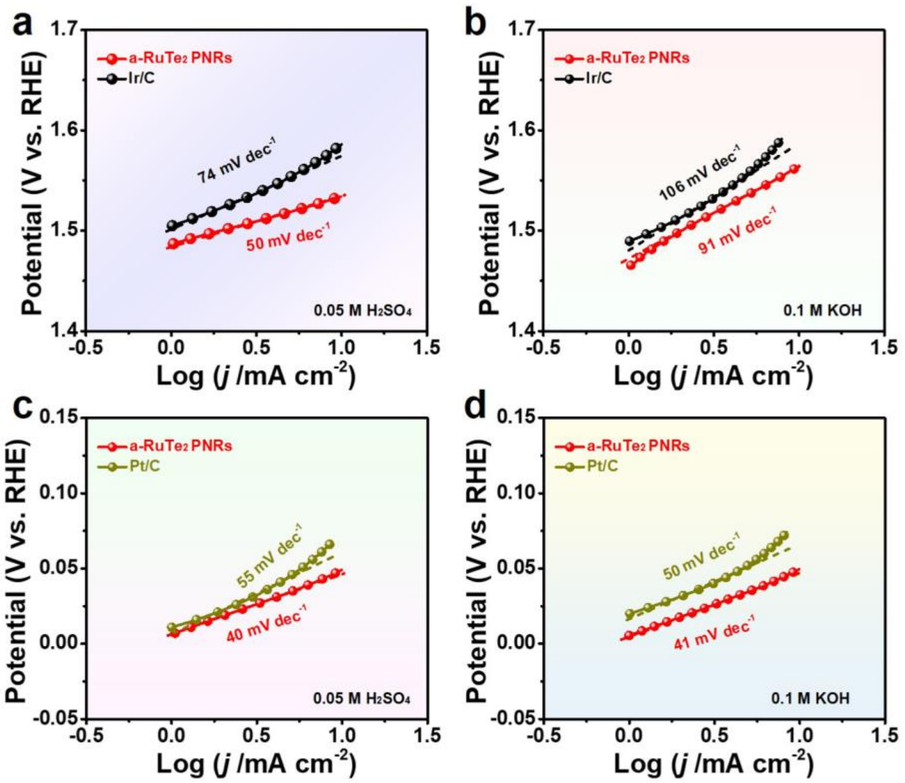

Supplementary Figure 10. OER Tafel slopes of a-$RuTe_2$ PNRs and Ir/C derived from Supplementary Figure 7a, b in (a) 0.05 M H_2SO_4 and (b) 0.1 M KOH. HER Tafel slopes of a-$RuTe_2$ PNRs and Pt/C derived from Supplementary Figure 8a, b in (c) 0.05 M H_2SO_4 and (d) 0.1 M KOH.

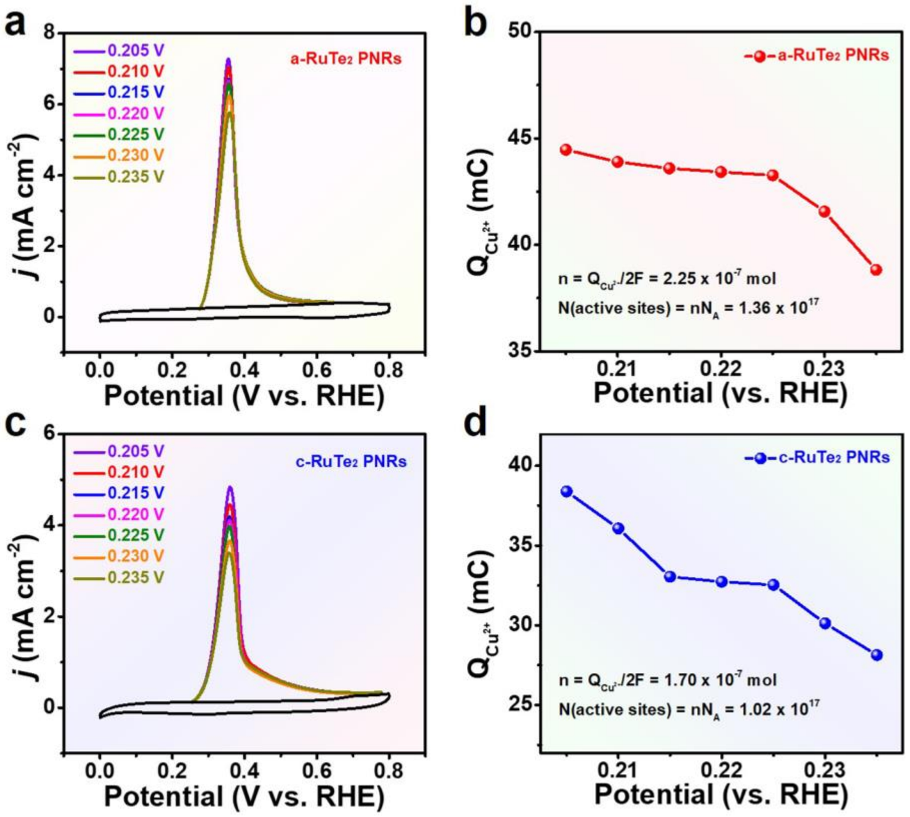

Supplementary Figure 11. (a, c) Cyclic voltammetry curves (black curves) of a-$RuTe_2$ PNRs and c-$RuTe_2$ PNRs in 0.5 M H_2SO_4 and polarization curves for the stripping of Cu deposited at different overpotentials from 0.205 to 0.235 V vs. RHE in a 0.5 M H_2SO_4 + 5 mM $CuSO_4$ solution for 100s to form the UPD layers on a-$RuTe_2$ PNRs and c-$RuTe_2$ PNRs. The charges required to strip the Cu deposited at different underpotentials for (b) a-$RuTe_2$ PNRs and (d) c-$RuTe_2$ PNRs. Scan rate: 10 mV s^{-1}.

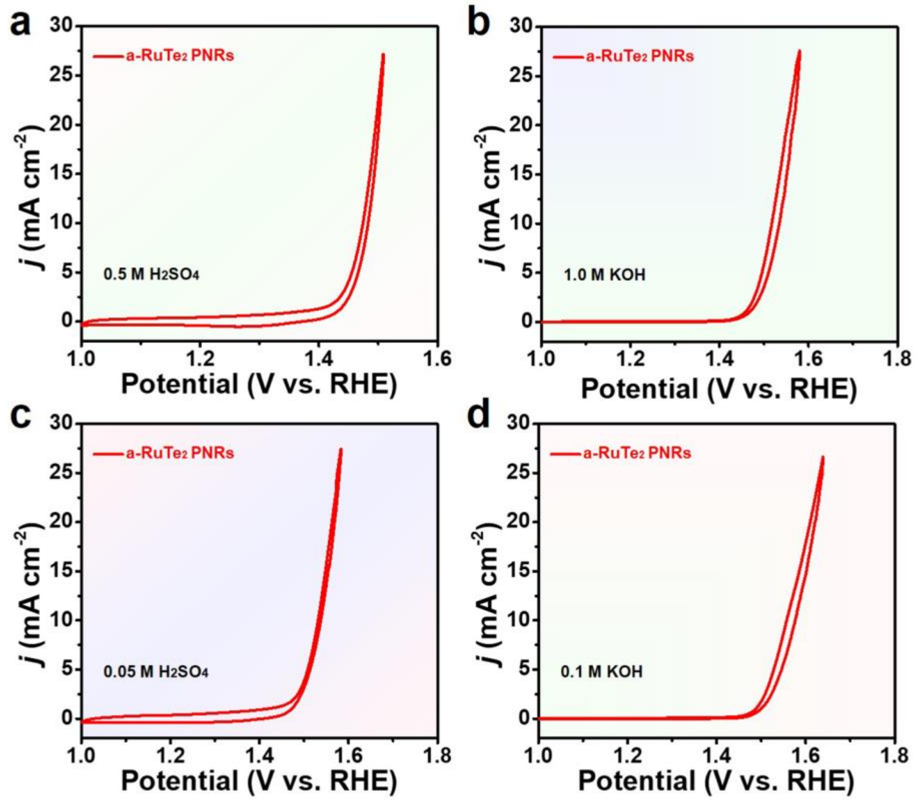

Supplementary Figure 12. Cyclic voltammetry curves of a-$RuTe_2$ PNRs in (a) 0.5 M H_2SO_4, (b) 1.0 M KOH, (c) 0.05 M H_2SO_4 and (d) 0.1 M KOH.

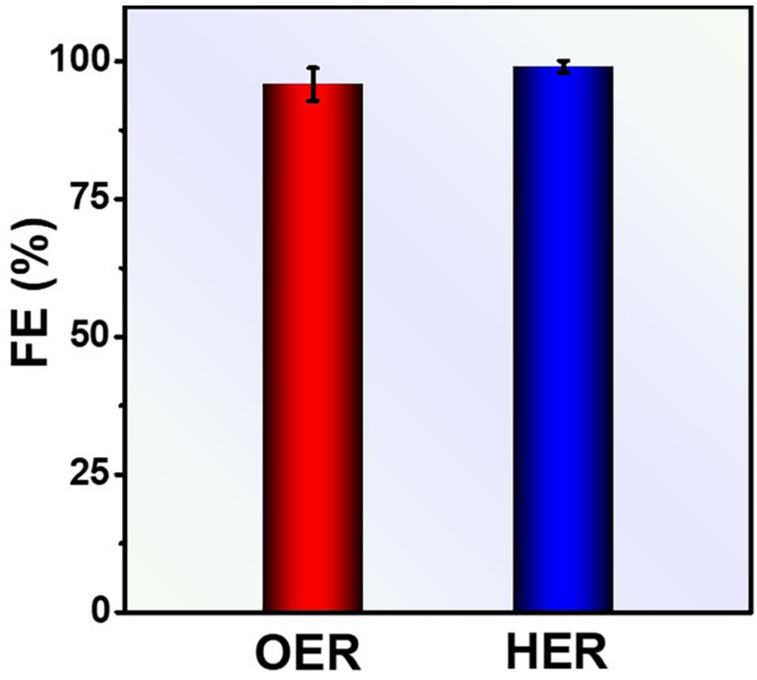

Supplementary Figure 13. Faraday efficiency of OER and HER for a-$RuTe_2$ PNRs in 0.5 M H_2SO_4.

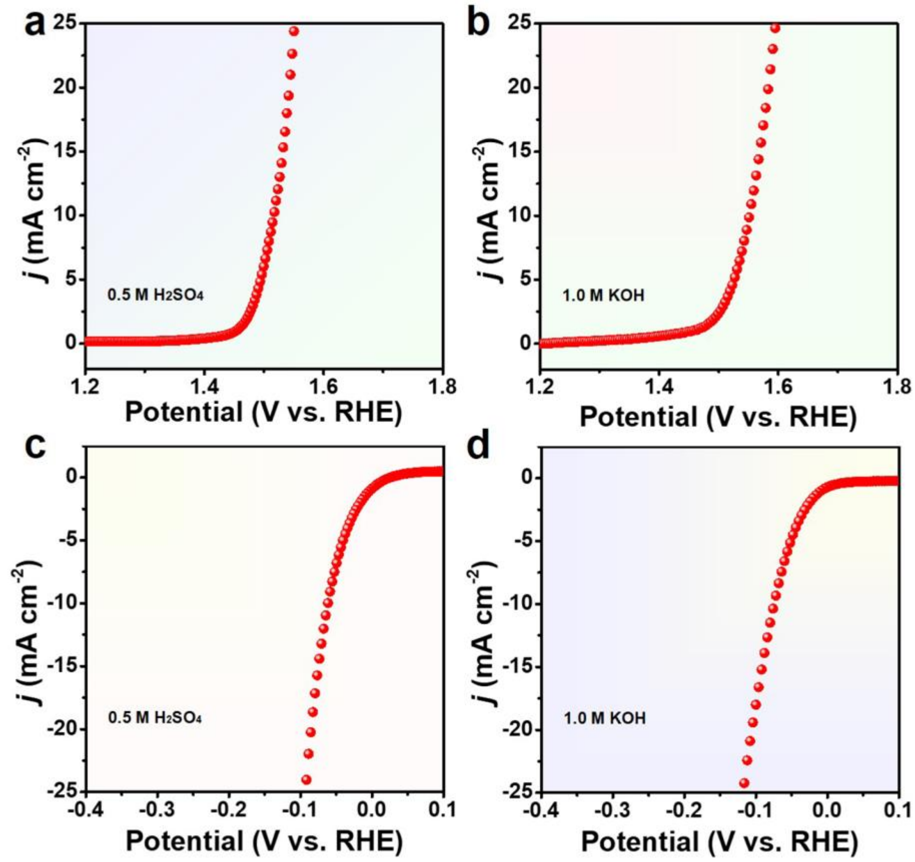

Supplementary Figure 14. HER and OER polarization curves of a-$RuTe_2$ PNRs without iR correction in (a, c) 0.5M H_2SO_4 and (b, d) 1.0 M KOH.

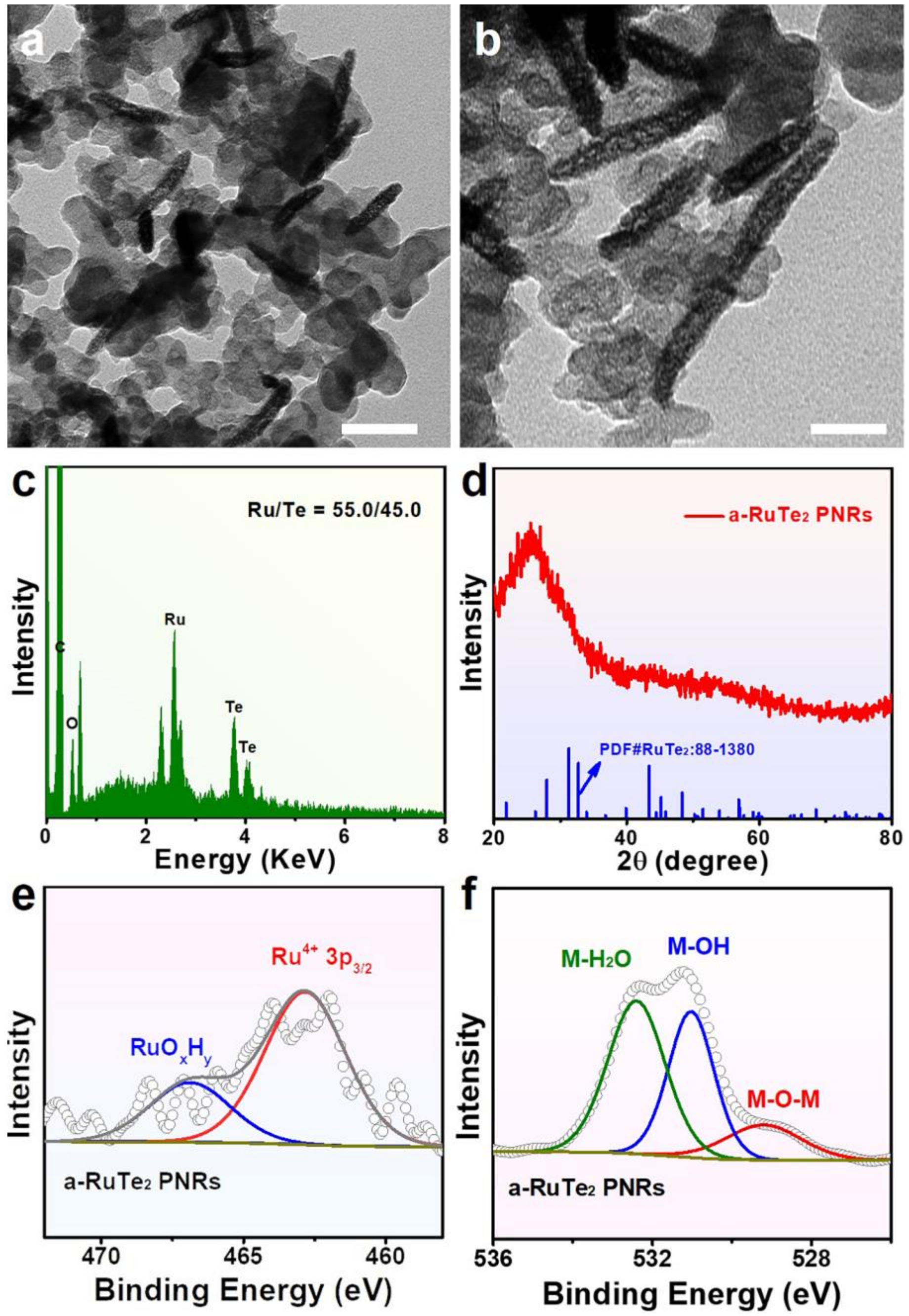

Supplementary Figure 15. (a, b) TEM images, (c) SEM-EDS spectrum, (d) PXRD pattern, (e) Ru 3p and (f) O 1s XPS spectra of a-$RuTe_2$ PNRs after water splitting (anode: OER) in 0.5 M H_2SO_4. Scale bar: (a) 100 nm; (b) 50 nm.

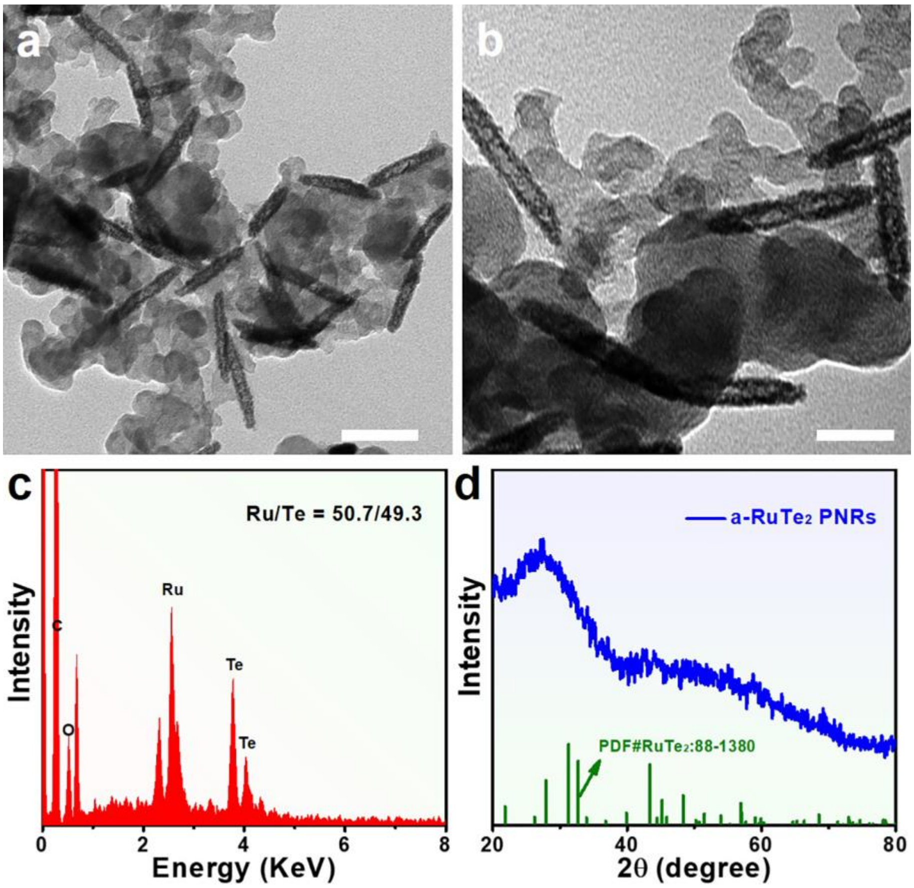

Supplementary Figure 16. (a, b) TEM images, (c) SEM-EDS spectrum, (d) PXRD pattern of a-$RuTe_2$ PNRs after water splitting (cathodic: HER) in 0.5 M H_2SO_4. Scale bar: (a) 100 nm; (b) 50 nm.

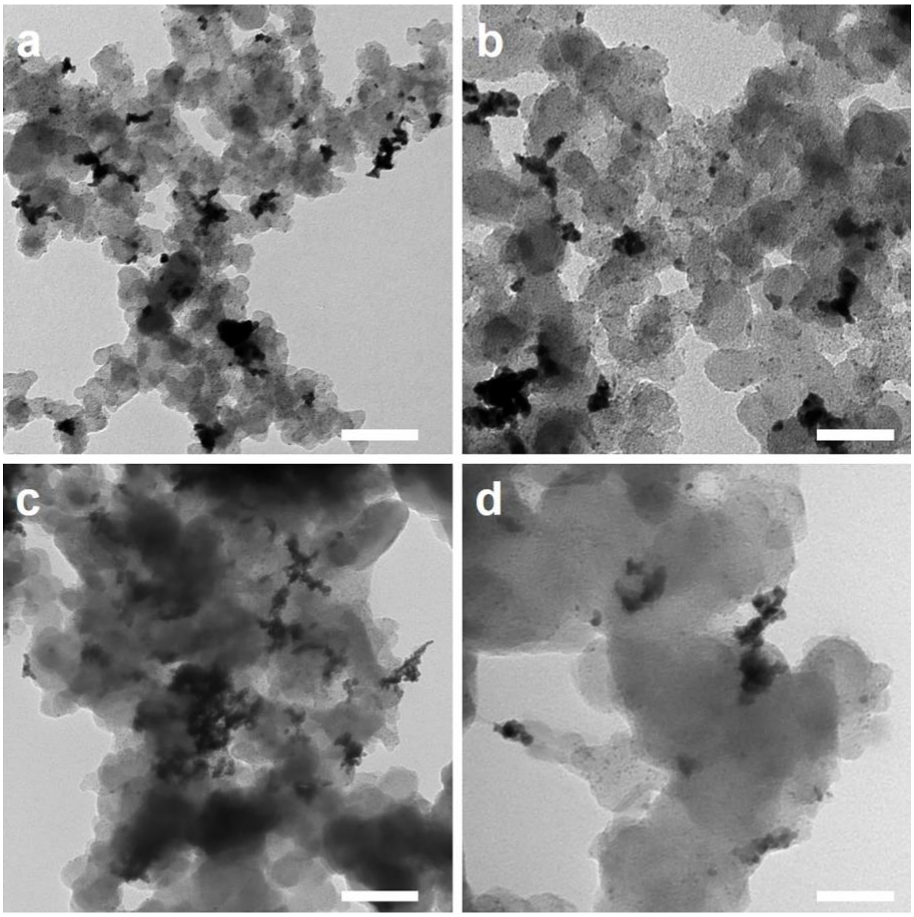

Supplementary Figure 17. TEM images of commercial Ir/C (a, b) before and (c, d) after water splitting in 0.5 M H_2SO_4. Scale bar: (a, c) 100 nm; (b, d) 50 nm.

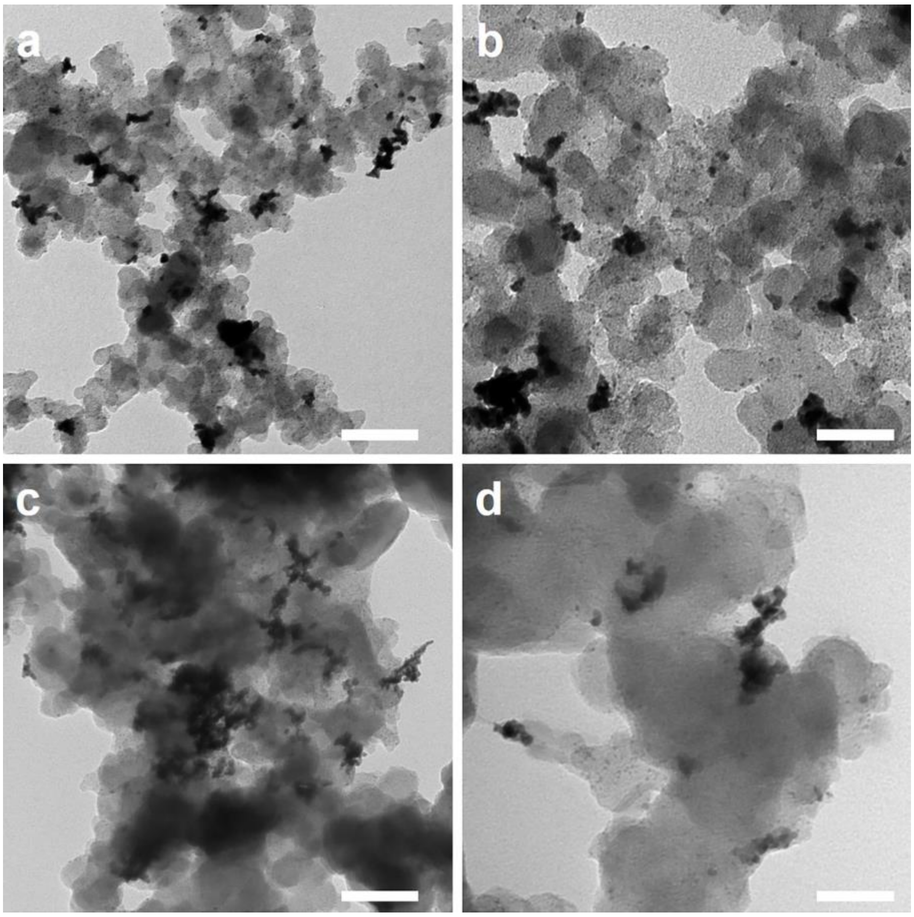

Supplementary Figure 18. TEM images of commercial Pt/C (a, b) before and (c, d) after water splitting in 0.5 M H_2SO_4. Scale bar: (a, c) 100 nm; (b, d) 50 nm.

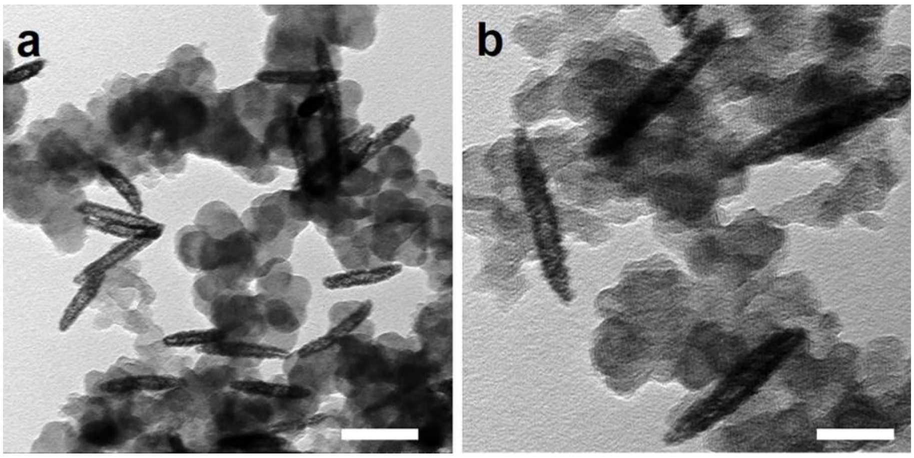

Supplementary Figure 19. TEM images of a-$RuTe_2$ PNRs after acidic treatment in 5.0 M H_2SO_4 at 60°C for 1h. Scale bar: (a) 100 nm; (b) 50 nm.

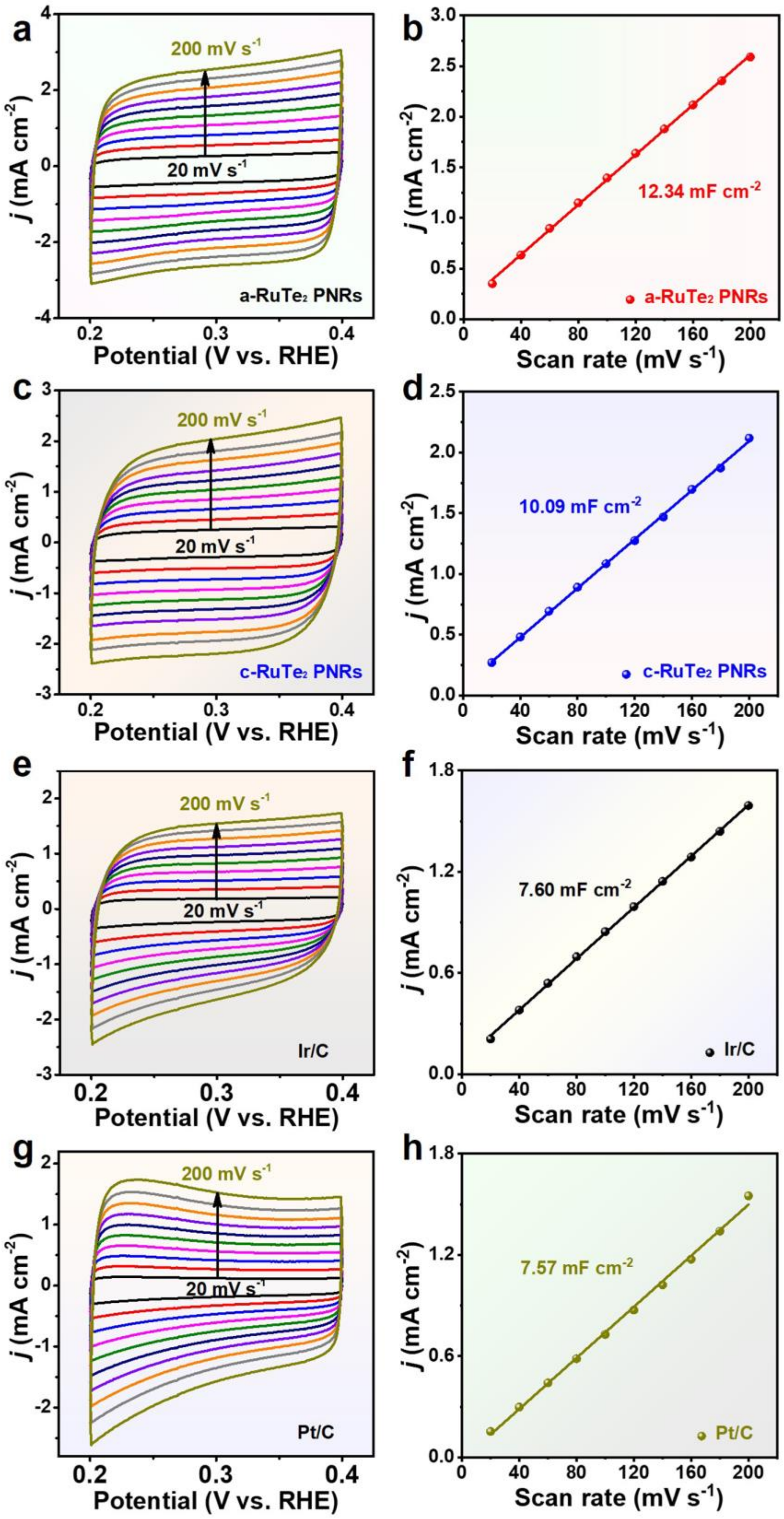

Supplementary Figure 20. (a, c, e, g) Cyclic voltammograms and (b, d, f, h) corresponding double layer capacitance (C_{dl}) of (a, b) a-$RuTe_2$ PNR, (c, d) c-$RuTe_2$ PNR, (e, f) Ir/C and (g, h) Pt/C.

Supplementary Table 1. Summary of reported OER electrocatalysts in different electrolytes.

Catalyst	Electrolyte	Current density	Overpotential (mV)	Reference
**a-$RuTe_2$ PNRs**	**0.5 M H_2SO_4**	**10 mA cm^{-2}**	**245**	**This work**
	**1.0 M KOH**		**285**	
$IrO_x/SrIrO_3$	0.5 M H_2SO_4	10 mA cm^{-2}	270	1
La_2LiIrO_6	0.5 M H_2SO_4	10 mA cm^{-2}	~300	2
Li-IrO_x	0.5 M H_2SO_4	10 mA cm^{-2}	300	3
Ni-NHGF	1.0 M KOH	10 mA cm^{-2}	330	4
Macro-TpBpy-Co	0.1 M KOH	10 mA cm^{-2}	380	5
TpBpy-Co			430	
$IrNi_2$-PE(H^+)	0.05 M H_2SO_4	10 mA cm^{-2}	315	6
$IrNi_2$-PE(OH^-)	0.1 M KOH	6.2 mA cm^{-2}	320	
Rh_2P/C	0.5 M H_2SO_4	5 mA cm^{-2}	510	7
$FeCoNiO_x$	1.0 M KOH	2 mA cm^{-2}	~260	8
$IrNiO_x$/Meso-ATO-180	0.5 M H_2SO_4	10 mA cm^{-2}	~320	9
Electrochemically formed IrO_x	0.1 M $HClO_4$	5 mA cm^{-2}	320	10
IrNi NCs	0.1 M $HClO_4$	10 mA cm^{-2}	280	11
FeCo-N_x-CN	1.0 M KOH	10 mA cm^{-2}	370	12
Ru	1.0 M H_2SO_4	10 mA cm^{-2}	340	13
	1.0 M KOH		320	
Ir	1.0 M H_2SO_4		360	
	1.0 M KOH		430	
NSFLGDT-900	1.0 M KOH	10 mA cm^{-2}	299	14
	0.1 M KOH		305	
6H-$SrIrO_3$	0.5 M H_2SO_4	10 mA cm^{-2}	248	15

NiCo-UMOFNs	1.0 M KOH	10 mA cm^{-2}	250	16
3D-Co	0.1 M KOH	10 mA cm^{-2}	429	17
2D-Co-NS			310	
Fe:2D-Co-NS			282	
Ir-STO	0.1 M $HClO_4$	10 mA cm^{-2}	247	18
LFNO-II-NRs	1.0 M KOH	10 mA cm^{-2}	302	19
dtf-IrOs	0.1 M $HClO_4$	10 mA cm^{-2}	300	20
Ni-P porous nanoplates	1.0 M KOH	10 mA cm^{-2}	300	21
h-PNRO/C	0.1 M $HClO_4$	10 mA cm^{-2}	239	22
γ-MnO_2	1.0 M H_2SO_4	10 mA cm^{-2}	489	23
$Ir_{0.7}Ru_{0.3}O_x$	0.05 M H_2SO_4	100 A a^{-1}	270	24
Ru@IrO_x	0.05 M H_2SO_4	10 mA cm^{-2}	282	25

Supplementary Table 2. Summary of reported HER electrocatalysts in different electrolytes.

**Catalyst**	**Electrolyte**	**Current density**	**Overpotential (mV)**	**Reference**
**a-$RuTe_2$ PNRs**	**0.5 M H_2SO_4**	**10 mA cm^{-2}**	**33**	**This work**
	**1.0 M KOH**		**36**	
SANi-PyNWs	1.0 M KOH	10 mA cm^{-2}	70	26
IFONFs-45	1.0 M KOH	10 mA cm^{-2}	47	27
Ru@CN-0.16	0.5 M H_2SO_4	10 mA cm^{-2}	126	28
	1.0 M KOH		32	
RuP_2@NPC	0.5 M H_2SO_4	10 mA cm^{-2}	38	29
	1.0 M KOH		52	
A-Ni@DG	0.5 M H_2SO_4	10 mA cm^{-2}	70	30
	1.0 M KOH		150	

CoMoNiS-NF-31	1.0 M KOH	5 mA cm^{-2}	77	31
Ru_2Ni_2 SNs/C	1.0 M KOH	10 mA cm^{-2}	40	32
N-$NiCo_2S_4$	1.0 M KOH	10 mA cm^{-2}	41	33
W-SAC	0.5 M H_2SO_4	10 mA cm^{-2}	105	34
	0.1 M KOH		85	
Ru/C_3N_4/C	0.5 M H_2SO_4	10 mA cm^{-2}	~70	35
	0.1 M KOH		79	
Co_1/PCN	1.0 M KOH	10 mA cm^{-2}	89	36
Rh-MoS_2	0.5 M H_2SO_4	10 mA cm^{-2}	47	37
Li-PPS NDs	0.5 M H_2SO_4	10 mA cm^{-2}	91	38
Ni@Ni_2P-Ru HNRs	0.5 M H_2SO_4	10 mA cm^{-2}	51	39
A-CoPt-NC	0.5 M H_2SO_4	10 mA cm^{-2}	27	40
	1.0 M KOH		50	
Rh/Si	0.5 M H_2SO_4	50 mA cm^{-2}	110	41
Co-Mo-S_x chalcogels	0.1 M KOH	5 mA cm^{-2}	220	42
Pt_1/OLC	0.5 M H_2SO_4	10 mA cm^{-2}	38	43
R-TiO2:Ru(5%)	0.1 M KOH	10 mA cm^{-2}	150	44
sc-$Ni_2P_{\delta-}$/NiHO	1.0 M KOH	10 mA cm^{-2}	60	45
Sr_2RuO_4	1.0 M KOH	10 mA cm^{-2}	61	46
Pt_3Ni_2-NWs-S/C	1.0 M KOH	10 mA cm^{-2}	42	47
	0.1 M KOH		45	
Ni-BDT-A	1.0 M KOH	10 mA cm^{-2}	80	48
EBP@NG(1:4)	1.0 M KOH	10 mA cm^{-2}	1125	49
Pt_3Ni frames/$Ni(OH)_2$/C	0.1 M KOH	4 mA cm^{-2}	~60	50

Supplementary Table 3. Summary of reported overall water splitting electrocatalysts in different electrolytes.

Catalyst	Electrolyte	Current density	Potential (V)	Reference
**a-$RuTe_2$ PNRs||a-$RuTe_2$ PNRs**	**0.5 M H_2SO_4**	**10 mA cm^{-2}**	**1.52**	**This work**
IrCoNi PHNCs||IrCoNi PHNCs	0.5 M H_2SO_4	4.66 A cm^{-2}	1.60	51
Ir_6Ag_9 NTs/C||Ir_6Ag_9 NTs/C	0.5 M H_2SO_4	10 mA cm^{-2}	1.55	52
IrNi NCs||IrNi NCs	0.5 M $HClO_4$	10 mA cm^{-2}	1.58	11
h-PNRO||h-PNRO	0.1 M $HClO_4$	10 mA cm^{-2}	1.52	22
ONPPGC/OCC||ONPPGC/OCC	0.5 M H_2SO_4	10 mA cm^{-2}	1.66	53
Ir/GF||Ir/GF	0.5 M H_2SO_4	10 mA cm^{-2}	1.55	54
Co-RuIr||Co-RuIr	0.1 M $HClO_4$	10 mA cm^{-2}	1.52	55
$IrNi_{0.57}Fe_{0.82}$||$IrNi_{0.57}Fe_{0.82}$	0.5 M $HClO_4$	10 mA cm^{-2}	1.64	56
Co/CoP||Co/CoP	0.5 M H_2SO_4	1 mA cm^{-2}	~1.90	57
NC-CNT/CoP||NC-CNT/CoP	0.5 M H_2SO_4	10 mA cm^{-2}	1.66	58
S-$NiFe_2O_4$/NF||S-$NiFe_2O_4$/NF	1 M PBS	10 mA cm^{-2}	1.95	59
$Ni_{0.1}Co_{0.9}P$||$Ni_{0.1}Co_{0.9}P$	1 M PBS	10 mA cm^{-2}	1.89	60
NiFe-MOF/NF||NiFe-MOF/NF	0.1 M KOH	10 mA cm^{-2}	1.55	61
CoP/NCNHP||CoP/NCNHP	1.0 M KOH	10 mA cm^{-2}	1.64	62
MoS_2/NiFe LDH|| MoS_2/NiFe LDH	1.0 M KOH	10 mA cm^{-2}	1.57	63
NiS_2/CoS_2||NiS_2/CoS_2	1.0 M KOH	10 mA cm^{-2}	1.78	64
Co_3O_4-MTA||Co_3O_4-MTA	1.0 M KOH	10 mA cm^{-2}	1.53	65
Ni-Co-P HNBs||Ni-Co-P HNBs	1.0 M KOH	10 mA cm^{-2}	1.60	66
Co_1Mo_1CH/NF||Co_1Mo_1CH/NF	1.0 M KOH	10 mA cm^{-2}	1.68	67
IFONFs-45||IFONFs-45	1.0 M KOH	10 mA cm^{-2}	1.58	27
Ir_1@Co/NC||Ir_1@Co/NC	1.0 M KOH	10 mA cm^{-2}	1.60	68

Co/β-Mo_2C@N-CNTs‖Co/β-Mo_2C@N-CNTs	1.0 M KOH	10 mA cm^{-2}	1.64	69
Ru_2Ni_2 SNs/C‖Ru_2Ni_2 SNs/C	1.0 M KOH	10 mA cm^{-2}	1.58	32
NiFe LDH/Ni foam‖ NiFe LDH/Ni foam	1.0 M NaOH	10 mA cm^{-2}	1.70	70

Supplementary Table 4. Positron lifetime parameters of a-$RuTe_2$ PNRs and c-$RuTe_2$ PNRs.

**Sample**	**a-$RuTe_2$ PNRs**	**c-$RuTe_2$ PNRs**	**Ir/C**	**Pt/C**
**C_{dl} /mF cm^2**	12.34	10.09	7.60	7.57
**ECSA /cm^2**	308.50	252.25	190.0	189.25

Supplementary Table 5. Positron lifetime parameters of a-$RuTe_2$ PNRs and c-$RuTe_2$ PNRs.

**Sample**	**τ_1 (ps)**	**I_1(%)**	**τ_2 (ps)**	**I_2(%)**
**a-$RuTe_2$ PNRs**	207.7	12.8	386.2	86.2
**c-$RuTe_2$ PNRs**	190.1	7.1	383.0	91.9

Supplementary References

1. Seitz, L. C. et al. A highly active and stable $IrO_x/SrIrO_3$ catalyst for the oxygen evolution reaction. *Science* **353**, 1011-1014 (2016).

2. Grimaud, A., Demortière, A., Saubanère, M., Dachraoui, W., Duchamp, M., Doublet, M. L. & Tarascon, J. M. Activation of surface oxygen sites on an iridium-based model catalyst for the oxygen evolution reaction. *Nat. Energy* **2**, 16189 (2016).

3. Gao, J. et al. Breaking long-range order in iridium oxide by alkali ion for efficient water oxidation. *J. Am. Chem. Soc.* **141**, 3014-3023 (2019).

4. Fei, H. et al. General synthesis and definitive structural identification of MN_4C_4 single-atom catalysts with tunable electrocatalytic activities. *Nat. Catal.* **1**, 63-72 (2018).

5. Zhao, X. et al. Macro/microporous covalent organic frameworks for efficient electrocatalysis. *J. Am. Chem. Soc.* **141**, 6623-6630 (2019).

6. Pi, Y., Shao, Q., Zhu, X., & Huang, X. Dynamic structure evolution of composition segregated iridium-nickel rhombic dodecahedra toward efficient oxygen evolution electrocatalysis. *ACS Nano* **12**, 7371-7379 (2018).

7. Duan, H. et al. High-performance Rh_2P electrocatalyst for efficient water splitting. *J. Am. Chem. Soc.* **139**, 5494-5502 (2017).

8. Smith, R. D. L. et al. Photochemical route for accessing amorphous metal oxide materials for water oxidation catalysis. *Science* **340**, 60-63 (2013).

9. Nong, H. N. et al. Oxide-supported IrNiOx core-shell particles as efficient, cost-effective, and stable catalysts for electrochemical water splitting. *Angew. Chem. Int. Ed.* **54**, 2975-2979 (2015).

10. Li, T. et al. Atomic-scale insights into surface species of electrocatalysts in three dimensions. *Nat. Catal.* **1**, 300-305 (2018).

11. Pi, Y., Shao, Q., Wang, P., Guo, J. & Huang, X. General formation of monodisperse IrM (M= Ni, Co, Fe) bimetallic nanoclusters as bifunctional electrocatalysts for acidic overall water splitting. *Adv. Funct. Mater.* **27**, 1700886 (2017).

12. Li, S., Cheng, C., Zhao, X., Schmidt, J. & Thomas, A. Active salt/silica-templated 2D mesoporous FeCo-Nx-carbon as bifunctional oxygen electrodes for zinc-air batteries. *Angew. Chem. Int. Ed.* **57**, 1856-1862 (2018).

13. McCrory, C. C. L., Jung, S., Ferrer, I. M., Chatman, S. M., Peters, J. C. & Jaramillo, T. F. Benchmarking hydrogen evolving reaction and oxygen evolving reaction electrocatalysts for solar water splitting devices. *J. Am. Chem. Soc.* **137**, 4347-4357 (2015).

14. Zhao, Y. et al. Stereodefined codoping of sp-N and S atoms in few-layer graphdiyne for oxygen evolution reaction. *J. Am. Chem. Soc.* **141**, 7240-7244 (2019).

15. Yang, L. et al. Efficient oxygen evolution electrocatalysis in acid by a perovskite with face-sharing IrO_6 octahedral dimers. *Nat. Commun.* **9**, 5236 (2018).

16. Zhao, S. et al. Ultrathin metal-organic framework nanosheets for electrocatalytic oxygen evolution. *Nat. Energy* **1**, 16184 (2016).

17. Huang, J. et al. Electrochemical exfoliation of pillared-layer metal-organic framework to boost the oxygen evolution reaction. *Angew. Chem. Int. Ed.* **130**, 4722-4726 (2018).

18. Liang, X. et al. Activating inert, nonprecious perovskites with iridium dopants for efficient oxygen evolution reaction under acidic conditions. *Angew. Chem. Int. Ed.* **58**, 7631-7635 (2019).

19. Wang, H., Wang, J., Pi, Y., Shao, Q., Tan, Y. & Huang, X. Double perovskite $LaFe_xNi_{1-x}O_3$ nanorods enable efficient oxygen evolution electrocatalysis. *Angew. Chem. Int. Ed.* **58**, 2316-2320 (2019).

20. Kim, Y. T. et al. Balancing activity, stability and conductivity of nanoporous core-shell iridium/iridium oxide oxygen evolution catalysts. *Nat. Commun.* **8**, 1449 (2017).

21. Yu, X. Y., Feng, Y., Guan, B., Lou, X. W. & Paik, U. Carbon coated porous nickel phosphides nanoplates for highly efficient oxygen evolution reaction. *Energy Environ. Sci.* **9**, 1246-1250 (2016).

22. Oh, A., Kim, H. Y., Baik, H., Kim, B., Chaudhari, N. K., Joo, S. H. & Lee, K. Topotactic transformations in an icosahedral nanocrystal to form efficient water-splitting catalysts. *Adv. Mater.* **31,** 1805546 (2018).

23. Li, A. et al. Stable potential windows for long-term electrocatalysis by manganese oxides under acidic

conditions. *Angew. Chem. Int. Ed.* **131,** 5108-5112 (2019).

24. Wang, L. et al. Highly active anode electrocatalysts derived from electrochemical leaching of Ru from metallic $Ir_{0.7}Ru_{0.3}$ for proton exchange membrane electrolyzers. *Nano Energy* **34**, 385-391 (2017).

25. Shan, J. et al. Charge-redistribution-enhanced nanocrystalline Ru@IrO_x electrocatalysts for oxygen evolution in acidic media. *Chem* **5**, 445-459 (2019).

26. Li, M. et al. Single-atom tailoring of platinum nanocatalysts for high-performance multifunctional electrocatalysis. *Nat. Catal.* **2**, 495-503 (2019).

27. Fan, X. et al. Defect-enriched iron fluoride-oxide nanoporous thin films bifunctional catalyst for water splitting. *Nat. Commun.* **9**, 1809 (2018).

28. Wang, J., Wei, Z., Mao, S., Li, H. & Wang, Y. Highly uniform Ru nanoparticles over N-doped carbon: pH and temperature-universal hydrogen release from water reduction. *Energy Environ. Sci.* **11**, 800-806 (2018).

29. Pu, Z., Amiinu, I. S., Kou, Z., Li, W. & Mu, S. RuP_2-based catalysts with platinum-like activity and higher durability for the hydrogen evolution reaction at all pH values. *Angew. Chem. Int. Ed.* **56**, 11559-11564 (2017).

30. Zhang, L. et al. Graphene defects trap atomic Ni species for hydrogen and oxygen evolution reactions. *Chem* **4**, 285-297 (2018).

31. Yang, Y. et al. Hierarchical nanoassembly of $MoS_2/Co_9S_8/Ni_3S_2$/Ni as a highly efficient electrocatalyst for overall water splitting in a wide pH range. *J. Am. Chem. Soc.* **141**, 10417-10430 (2019).

32. Ding, J., Shao, Q., Feng, Y. & Huang, X. Ruthenium-nickel sandwiched nanoplates for efficient water splitting electrocatalysis. *Nano Energy* **47**, 1-7 (2018).

33. Wu, Y. et al. Electron density modulation of $NiCo_2S_4$ nanowires by nitrogen incorporation for highly efficient hydrogen evolution catalysis. *Nat. Commun.* **9**, 1425 (2018).

34. Chen, W. et al. Single tungsten atoms supported on MOF-derived N-doped carbon for robust electrochemical

hydrogen evolution. *Adv. Mater.* **30**, 1800396 (2018).

35. Zheng, Y. et al. High electrocatalytic hydrogen evolution activity of an anomalous ruthenium catalyst. *J. Am. Chem. Soc.* **138**, 16174-16181 (2016).

36. Cao, L. et al. Identification of single-atom active sites in carbon-based cobalt catalysts during electrocatalytic hydrogen evolution. *Nat. Catal.* **2**, 134-141 (2018).

37. Cheng, Y., Lu, S., Liao, F., Liu, L., Li, Y. & Shao, M. Rh-MoS_2 nanocomposite catalysts with Pt-like activity for hydrogen evolution reaction. *Adv. Funct. Mater.* **27**, 1700359 (2017).

38. Zhang, X. et al. Lithiation-induced amorphization of $Pd_3P_2S_8$ for highly efficient hydrogen evolution. *Nat. Catal.* **1**, 460-468 (2018).

39. Liu, Y. et al. Ru modulation effects in the synthesis of unique rod-like Ni@Ni_2P-Ru heterostructures and their remarkable electrocatalytic hydrogen evolution performance. *J. Am. Chem. Soc.* **140**, 2731-2734 (2018).

40. Zhang, L. et al. Charge polarization from atomic metals on adjacent graphitic layers for enhancing the hydrogen evolution reaction. *Angew. Chem. Int. Ed.* **58**, 9404-9408 (2019).

41. Zhu, L. et al. A rhodium/silicon co-electrocatalyst design concept to surpass platinum hydrogen evolution activity at high overpotentials. *Nat. Commun.* **7**, 12272 (2016).

42. Staszak-Jirkovský, J. et al. Design of active and stable Co-Mo-S_x chalcogels as pH-universal catalysts for the hydrogen evolution reaction. *Nat. Mater.* **15**, 197-203 (2016).

43. Liu, D. et al. Atomically dispersed platinum supported on curved carbon supports for efficient electrocatalytic hydrogen evolution. *Nat. Energy* **4**, 512-518 (2019).

44. Nong, S. et al. Well-dispersed ruthenium in mesoporous crystal TiO_2 as an advanced electrocatalyst for hydrogen evolution reaction. *J. Am. Chem. Soc.* **140**, 5719-5727 (2018).

45. You, B., Zhang, Y., Jiao, Y., Davey, K. & Qiao, S. Z. Negative charging of transition-metal phosphides via

strong electronic coupling for destabilization of alkaline water. *Angew. Chem. Int. Ed.* **58**, 11796-11800 (2019).

46. Zhu, Y. et al. Unusual synergistic effect in layered Ruddlesden-popper oxide enables ultrafast hydrogen evolution. *Nat. Commun.* **10**, 149 (2019).

47. Wang, P. et al. Precise tuning in platinum-nickel/nickel sulfide interface nanowires for synergistic hydrogen evolution catalysis. *Nat. Commun.* **8**, 14580 (2017).

48. Hu, C. et al. In situ electrochemical production of ultrathin nickel nanosheets for hydrogen evolution electrocatalysis. *Chem* **3**, 122-133 (2017).

49. Yuan, Z. et al. Ultrathin black phosphorus-on-nitrogen doped graphene for efficient overall water splitting: dual modulation roles of directional interfacial charge transfer. *J. Am. Chem. Soc.* **141**, 4972-4979 (2019).

50. Chen, C. et al. Highly crystalline multimetallic nanoframes with three-dimensional electrocatalytic surfaces. *Science* **343**, 1339-1343 (2014).

51. Feng, J. et al. Iridium-based multimetallic porous hollow nanocrystals for efficient overall-water-splitting catalysis. *Adv. Mater.* **29**, 1703798 (2017).

52. Zhu, M., Shao, Q., Qian, Y. & Huang, X. Superior overall water splitting electrocatalysis in acidic conditions enabled by bimetallic Ir-Ag nanotubes. *Nano Energy* **56**, 330-337 (2019).

53. Lai, J., Li, S., Wu, F., Saqib, M., Luque, R. & Xu, G. Unprecedented metal-free 3D porous carbonaceous electrodes for full water splitting. *Energy Environ. Sci.* **9**, 1210-1214 (2016).

54. Zhang, J. et al. Iridium nanoparticles anchored on 3D graphite foam as a bifunctional electrocatalyst for excellent overall water splitting in acidic solution. *Nano Energy* **40**, 27-33 (2017).

55. Shan, J., Ling, T., Davey, K., Zheng, Y. & Qiao, S. Z. Transition-metal-doped RuIr bifunctional nanocrystals for overall water splitting in acidic environments. *Adv. Mater.* **31**, 1900510 (2019).

56. Fu, L., Cheng, G. & Luo, W. Colloidal synthesis of monodisperse trimetallic IrNiFe nanoparticles as highly

active bifunctional electrocatalysts for acidic overall water splitting. *J. Mater. Chem. A.* **5**, 24836-24841 (2017).

57. Xue, Z. H., Su, H., Yu, Q. Y., Zhang, B., Wang, H. H., Li, X. H. & Chen, J. S. Janus Co/CoP nanoparticles as efficient Mott-Schottky electrocatalysts for overall water splitting in wide pH range. *Adv. Energy Mater.* **7**, 1602355 (2017).

58. Guan, C. et al. Metal-organic framework-derived integrated nanoarrays for overall water splitting. *J. Mater. Chem. A* **6**, 9009-9018 (2018).

59. Liu, J., Zhu, D., Ling, T., Vasileff, A. & Qiao, S. Z. S-$NiFe_2O_4$ ultra-small nanoparticle built nanosheets for efficient water splitting in alkaline and neutral pH. *Nano Energy* **40**, 264-273 (2017).

60. Wu, R. et al. A janus nickel cobalt phosphide catalyst for high-efficiency neutral-pH water splitting. *Angew. Chem. Int. Ed.* **130**, 15671-15675 (2018).

61. Duan, J., Chen, S. & Zhao, C. Ultrathin metal-organic framework array for efficient electrocatalytic water splitting. *Nat. Commun.* **8**, 15341 (2017).

62. Pan, Y. et al. Core-shell ZIF-8@ZIF-67-derived CoP nanoparticle-embedded N-doped carbon nanotube hollow polyhedron for efficient overall water splitting. *J. Am. Chem. Soc.* **140**, 2610-2618 (2018).

63. Xiong, P. et al. Interface modulation of two-dimensional superlattices for efficient overall water splitting. *Nano Lett.* **19**, 4518-4526 (2019).

64. Yin, J. et al. Oxygen vacancies dominated NiS_2/CoS_2 interface porous nanowires for portable Zn-Air batteries driven water splitting devices. *Adv. Mater.* **29**, 1704681 (2017).

65. Zhu, Y. P., Ma, T. Y., Jaroniec, M. & Qiao, S. Z. Self-templating synthesis of hollow Co_3O_4 microtube arrays for highly efficient water electrolysis. *Angew. Chem. Int. Ed.* **56**, 1324-1328 (2017).

66. Hu, E., Feng, Y., Nai, J., Zhao, D., Hu, Y. & Lou, X. W. Construction of hierarchical Ni-Co-P hollow nanobricks with oriented nanosheets for efficient overall water splitting. *Energy Environ. Sci.* **11**, 872-880 (2018).

67. Tang, T. et al. Electronic and morphological dual modulation of cobalt carbonate hydroxides by Mn doping toward highly efficient and stable bifunctional electrocatalysts for overall water splitting. *J. Am. Chem. Soc.* **139**, 8320-8328 (2017).

68. Lai, W. H. et al. General π-electron-assisted strategy for Ir, Pt, Ru, Pd, Fe, Ni single-atom electrocatalysts with bifunctional active sites for highly efficient water splitting. *Angew. Chem. Int. Ed.* **131**, 11994-11999 (2019).

69. Ouyang, T., Ye, Y. Q., Wu, C. Y., Xiao, K. & Liu, Z. Q. Heterostructures composed of N-doped carbon nanotubes encapsulating cobalt and β-Mo_2C nanoparticles as bifunctional electrodes for water splitting. *Angew. Chem. Int. Ed.* **58**, 4923-4928 (2019).

70. Luo, J. et al. Water photolysis at 12.3% efficiency via perovskite photovoltaics and earth-abundant catalysts. *Science* **345**, 1593-1596 (2014).
